# The role of cortical oscillations in a spiking neural network model of the basal ganglia

**DOI:** 10.1371/journal.pone.0189109

**Published:** 2017-12-13

**Authors:** Zafeirios Fountas, Murray Shanahan

**Affiliations:** Department of Computing, Imperial College London, London, United Kingdom; University of California Los Angeles, UNITED STATES

## Abstract

Although brain oscillations involving the basal ganglia (BG) have been the target of extensive research, the main focus lies disproportionally on oscillations generated within the BG circuit rather than other sources, such as cortical areas. We remedy this here by investigating the influence of various cortical frequency bands on the intrinsic effective connectivity of the BG, as well as the role of the latter in regulating cortical behaviour. To do this, we construct a detailed neural model of the complete BG circuit based on fine-tuned spiking neurons, with both electrical and chemical synapses as well as short-term plasticity between structures. As a measure of effective connectivity, we estimate information transfer between nuclei by means of transfer entropy. Our model successfully reproduces firing and oscillatory behaviour found in both the healthy and Parkinsonian BG. We found that, indeed, effective connectivity changes dramatically for different cortical frequency bands and phase offsets, which are able to modulate (or even block) information flow in the three major BG pathways. In particular, alpha (8–12Hz) and beta (13–30Hz) oscillations activate the direct BG pathway, and favour the modulation of the indirect and hyper-direct pathways via the subthalamic nucleus—globus pallidus loop. In contrast, gamma (30–90Hz) frequencies block the information flow from the cortex completely through activation of the indirect pathway. Finally, below alpha, all pathways decay gradually and the system gives rise to spontaneous activity generated in the globus pallidus. Our results indicate the existence of a multimodal gating mechanism at the level of the BG that can be entirely controlled by cortical oscillations, and provide evidence for the hypothesis of cortically-entrained but locally-generated subthalamic beta activity. These two findings suggest new insights into the pathophysiology of specific BG disorders.

## Introduction

Rhythmic activity is one of the most widely-studied phenomena in the brain [1]. In the mammalian cortex, oscillations in low-frequency ranges (<100 Hz) have been associated with numerous cognitive and motor functions, that vary from feature binding [2] and mental simulation [3] to movement preparation and execution [4]. This cortical phenomenon provides a fruitful framework to study neural computation and has given rise to theories that account for the control of communication between regions [5, 6] as well as memory formation and retrieval [7].

Oscillatory phenomena are not only prevalent in the cortex but also a prominent feature of other sub-cortical structures. In the basal ganglia (BG), a fundamental structure of all vertebrate brains [8, 9], low-frequency oscillations are ubiquitous during spontaneous activity. Moreover, they are magnified in neurodegenerative disorders that affect this structure, such as Parkinson’s (PD) or Huntington’s (HD) disease. Although the cortex and the BG are highly interconnected, both functionally and structurally, it is still unclear which elements of this rich oscillatory behaviour are generated in the cortex and processed in the BG, or vice versa.

Experimental and theoretical studies have provided initial evidence suggesting that BG activity at some specific frequency bands is driven by areas of the cortex [10, 11]. Moreover, those signals appear to be not simply relayed through the BG pathways but are rather subjected to some sort of internal processing, depending on their initial frequency [11]. However, most of the evidence that has been acquired so far does not come from studies on healthy humans, due to the inability of most current non-invasive recording techniques to be applied in sub-cortical structures. Instead, most studies are confined either to animal models or human patients who undergo deep brain stimulation (DBS), a common surgical treatment of BG diseases which provides the opportunity to record the spiking activity of multiple structures simultaneously.

From a behavioural perspective, the BG are widely-assumed and recently found to be a key component in voluntary action selection and motor planning [12–15]. One of their roles is to provide reactive behavioural inhibition via competition between their main pathways. They have been also found to be involved in sequence learning [16] and working memory [17]. Moreover, a pathological disturbance of the balance between these pathways, for instance after the depletion of the neurotransmitter dopamine in PD, can cause a number of motor symptoms including tremor, bradykinesia and rigidity, as well as various cognitive and psychiatric dysfunctions [18].

For all the aforementioned reasons, a substantial number of computational models have been proposed [19–25] (for reviews see [26, 27]) in order to investigate BG pathophysiology and assess their role in signal processing, motor and cognitive control. Yet the topic of cortical oscillations is largely neglected in the majority of these models which, depending on their level of detail, focus either on inter- or intra-nuclei interactions and locally generated rhythms.

The purpose of this study is to redress this imbalance and foreground the theme of cortical oscillations by means of a new biologically plausible computational model of the BG circuitry. Our model is the first, to our knowledge, to integrate fine-tuned models of phenomenological spiking neurons, hence it is called neural, that correspond to different sub-types of cells within the BG nuclei, electrical and plastic chemical synapses and anatomically-derived striatal connectivity. Using this model, we carry out an analysis of the relationship between cortical frequency, level of dopamine, locally generated oscillations and the information flow between the BG structures.

We found that the effective connectivity between the BG substructures, and by extension BG function, is completely controlled by the frequency and phase of cortical oscillations. Via this mechanism, cortical signals can be relayed, blocked or transformed depending on which BG pathway remains open in each frequency range. Hence, information that has been processed in the cortex can either continue to reverberate through functionally-connected cortical regions, or flow through the thalamus. These results suggest that the BG can be viewed as the “gear box” of the cortex. Different rhythmic cortical areas are able to switch between a repertoire of available basal ganglia modes which, in turn, change the course of information flow back to and within the cortex. Furthermore, we predict that exaggerated beta band activity, a typical symptom in PD [11, 28], originates in the subthalamic nucleus (STN) on account of the dynamics of individual neurons within this structure, but it is entrained by the cortex.

The discussion of this paper provides a review of the literature related to low-frequency bands, along with a comparison against our results, which leads to a number of proposed cognitive mechanisms related to each band. Finally, we point out the impact of the phase offset between cortical oscillators on the interaction between the STN and the external segment of globus pallidus (GPe), as well as its role in modulating BG output.

## Results

### New BG neural model

**Fig 1:**
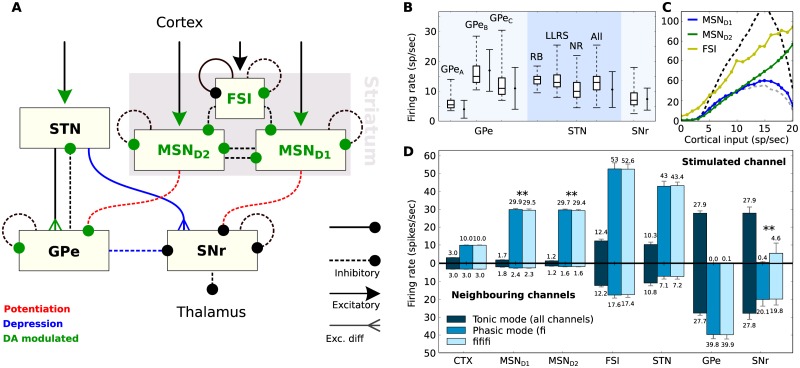
Architecture and firing behaviour of the system. **A**: The BG circuit as realized in the present study. Dopamine (DA) influences both the internal behaviour of MSNs and FSIs as well as the impact of various synaptic conductances. **B**: Firing rates of the various neuron types when isolated. Boxplots show median, first-third quartiles, and minimum-maximum of the mean firing rate of each simulated neuron. Solid error bars represent mean and standard deviation of recorded (real) spontaneous activity. Data for GPe neurons were taken from [30], for STN neurons from [31] and for SNr neurons from [32]. Neuron sub-types of the striatum include medium spiny-projection neurons (MSN) and fast-spiking interneurons (FSI), the STN includes rebound-bursting (RB), long-lasting rebound spikes (LLRS) and no-rebound (NR) neurons, and the GPe includes the three types GPe_*A*−*C*_. **C**: Cortical input-firing rate curve of striatal neurons when the complete model is in use. The dashed lines illustrate the MSN_*D*1_ curve for low dopamine (grey) and high dopamine (black) in the system. **D**: The mean firing rates, over 500 3-second trials, of the various neuron types when the complete model is in use. The *stimulated channel* represents the channel that received enhanced cortical input during the phasic mode, while the set of bar charts below show the firing rates of the two *neighbouring channels*. The error bars show standard deviation. In tonic mode, there is no discrimination between channels and the small differences in the two sets of bar charts are the result of random noise. The double asterisk (**) denotes an independent two-sample T-test with p-value < < 0.01.

Our optimization process resulted in eight new spiking models of BG neurons, based on the phenomenological Izhikevich equations [29], that were integrated into a large-scale model of the BG canonical circuit. This was successfully tuned to reproduce the firing patterns observed in biological BG neurons, both measured in brain slices as well as in *in vivo* behavioural studies. Fig 1.A shows the internal structure of the model, with emphasis on the synaptic types between the BG nuclei. The spontaneous firing rates of all optimized types of neurons, when their synaptic input current is zero, are compared with real data in Fig 1.B.

The optimization of the connectivity between BG nuclei was achieved based on two different functional scenarios that resulted in the firing rates illustrated in Fig 1.D, which will be termed as *tonic* and *phasic* modes throughout this document. In the tonic mode, the model received the same cortical input in all input areas, which had a low mean firing rate of 3 spikes/sec and represented the default tonically-active state of the BG neurons. Additionally, the phasic mode was accompanied by a higher level of stimulation in a single microscopic channel, the subgroup of BG neurons that are structurally connected to a particular cortical ensemble, via a fixed 10 spikes/sec-activation of the corresponding ensemble (see S1 Fig). This enhanced cortical input was able to cause transient effects in the BG network, and it represented the scenario that this part of the BG circuitry is highly engaged in a motor or cognitive task.

In both modes, the model produced behaviour which agrees well with the current literature. The phasic cortical stimulation of a single channel was enough to drop the firing rate of SNr to almost 0 spikes/sec, while activity in neighbouring channels decreased to only around 29%. This behaviour has been associated with decision making [13, 19, 33], since it allows the BG circuitry to selectively halt inhibition of the area in the thalamus that is targeted by the affected microscopic channel.

In additional experiments, the cortical input that the model received had an oscillatory behaviour, as described in methodology, with a mutual amplitude across the cortical spike generators of the same channel and a fixed frequency, picked randomly from 0 to 100Hz. In the case of a phasic channel, the amplitude of the oscillations was 10 spikes/sec while the amplitude in tonic channels was again 3 spikes/sec. Although this type of phasic input caused almost identical changes to the firing rates of the STN and GPe, compared to the initial experiments where each channel had a fixed firing rate, it had a strong influence on the activity of SNr, as well as some small influence on the striatum (see statistical tests in Fig 1.D). In particular, the SNr firing rate varied greatly for different cortical frequencies, between 0.13 and 23.71 spikes/sec compared to 0.07–1.25 spikes/sec in the static case, with a standard deviation of 6.33 spikes/sec. A Spearman’s rank-order correlation coefficient test between the cortical frequency and the firing rate of SNr resulted in *ρ* = −0.882 and p-value <10^−40^, indicating a nearly monotonic relationship. However, despite the fact that SNr showed such a different behaviour in the same channel, the firing rates of the neighbouring channels were indistinguishable in both cases (p-value of T-test: ∼0.569).

Finally, when given strong cortical stimulation, the model produced symmetric activity in both groups of MSN neurons, at around 30 spikes/sec. This was a result of the fine balance between MSN_*D*1_ excitation, which is potentiated by dopamine, and connectivity asymmetries in local inhibition favouring MSN_*D*2_ neurons. Further simulations revealed the existence of a transition threshold at around 9.5 spikes/sec of cortical stimulation, above which, the firing rate of MSN_*D*2_ neurons exceeds MSN_*D*1_, supporting the recently-proposed hypothesis of a decision threshold between the direct and indirect pathways in the striatum [34].

Fig 1.C illustrates this transition of the dominating neuron type, as well as the effect of dopamine in MSN_*D*1_ neurons that resulted in the modulation of the former. The baseline dopamine level is considered to be 30% [19, 23]. In low dopamine conditions (0%), this decision threshold shifts to around 3 spikes/sec of cortical stimulation while for high dopamine (90%), it exceeds 18 spikes/sec, an unrealistically high rate for corticostriatal neurons during behaviour [35].

### Dopaminergic modulation of intrinsically-induced beta oscillations in the GPe-STN loop

One major and well-studied feature of the BG function is the existence of strong, intrinsically-generated, oscillatory activity that originates from recurrent connections between the STN and GPe [36, 37]. The next step of this work was to investigate the oscillatory behaviour generated within our model, before moving to cortical oscillations, in order to assess the extent to which it agrees with the literature. To calculate the power spectra of the different BG structures we employed the multitaper method [38], which offers good frequency specificity and is able to detect low-frequency signals, better than other typical methods [39]. This method was applied on 1ms-binned, mean-centred and Gaussian-smoothed spike trains, with a simulated duration of 3 seconds each.

**Fig 2:**
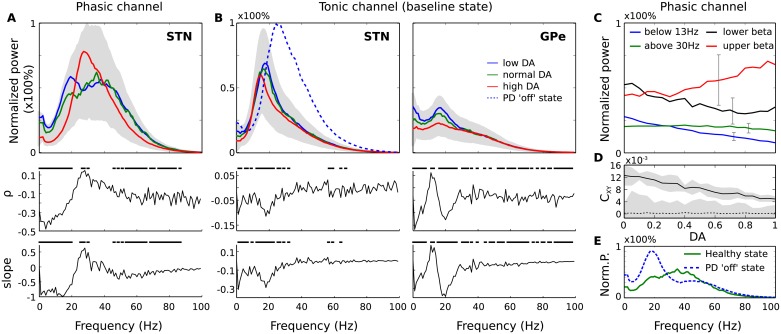
Frequency spectrum of STN-GPe loop without oscillatory input. Dopamine reduces lower-beta oscillations and modulates the spectrum. **A**, **B**: Up: Average power spectrum for different dopamine levels during the phasic (A) and tonic fixed (B) state of a single microscopic channel in the level of the STN and GPe. The spectrum is normalized by the maximum value of STN power in A, and both STN and GPe in B. The light grey areas represent the standard deviation of the average power spectrum for random dopamine levels between 0 and 1 (*n* = 4000). Down: Spearman’s rank correlation coefficient (*ρ*) and the slope of best-fit regression line between frequency power and DA levels measured in the above runs. The black lines indicate areas where p-value is less than 0.05. **C**: Mean power of the four interesting frequency bands for different plausible dopamine levels, during the phasic state of a microscopic channel in STN. **D**: Average cross-correlation between the firing rates of each neuron in the STN, presented for different levels of dopamine and used as a measure of synchrony within the STN. The solid line represents correlation when stimulation to the BG is applied transiently, after the subtraction of the same statistic produced by surrogate data, while the dashed line shows the result of stimulation over a long time period. **E**: Average power spectrum of a phasic STN channel under healthy and PD conditions. In all cases, the shaded areas or lines represent standard deviation.

Without any fluctuations of the firing rate of the input ensembles, the model was able to generate beta oscillations internally, mainly visible in STN and GPe, whose peak frequency varied depending on the activation of each channel. When the stimulation was limited at the tonic levels, the STN displayed strong lower-beta oscillations with a sharp peak at 18–20Hz while the GPe showed a weaker peak at the same frequencies (Fig 2.B). An increase of the input firing rate to 10 spikes/sec (phasic input) to a single channel, enough to cause silence in GPe and SNr, diminished the difference between areas of low frequency bands in STN (10–50Hz), which remained, however, highly active (Fig 2.A).

Furthermore, the level of dopamine in the neuron equations of the network was found to modulate these low-frequency oscillations in different ways. In an phasic STN channel, dopamine above the normal levels (*d*_1,2_ > 0.3) was able to suppress the power of oscillations lower than 20Hz (Fig 2.C) and strongly amplify the upper-beta band (23–30Hz), resulting in a clear peak at 28–30Hz. On the other hand, low dopamine caused an amplification of the lower-beta band, almost linearly proportional to the level of reduction, from 0% to 30%, without any significant effects on the other frequency bands.

Finally, in tonic activation of a BG channel, high levels of dopamine caused a slight shift in the peak beta frequency in STN and abolished any indication of enhanced beta activity in GPe. As in the case of a phasic channel, these oscillatory effects were more noticeable for dopamine values significantly higher than the net concentration. This dopamine increase is expected in healthy brains, where the level of dopamine can be boosted by phasic release during behaviour [40].

Interestingly, similar oscillatory patterns have been found in clinical recordings of PD patients, during “on-” and “off-medication” periods [41]. To simulate the ‘off’ Parkinsonian BG state more accurately, we assumed complete dopamine depletion (*d*_1,2_ = 0), as well as an increase of the cortical impact to the striatum and STN. Although PD does not influence the firing rate of the majority of biological corticostriatal neurons, low dopaminergic transmission has been shown to cause high levels of cross-correlated activity between the cortex and the striatum [42] and hyperactivity in STN [43, 44]. Hence, to capture this effect here, we tested two different adjustments to the model, a 20% increase of the cortical firing rate, as well as a 10% increase of the conductance of the synapses that originate from the cortical ensembles. Both simulations resulted in almost identical changes in STN behaviour, that agree well with the literature [41, 43]. These comprise a substantial increase of the power of lower-beta oscillations, shown in Fig 2.E, a 20% drop of low-gamma and upper-beta oscillations, and a 20% increase of the overall STN firing rate.

The excessively rhythmic behaviour of the phasic STN is particularly interesting, as its neurons remained uncoupled without the inhibition of GPe (most GPe neurons connected to a phasic STN channel remain silent), leading to the conclusion that the emergent oscillatory patterns are a result of membrane potential dynamics of the STN neurons.

In pursuit of this idea, we conducted a statistical analysis comparing the interspike intervals (ISIs) of the three simulated neuron types in STN, in order to evaluate the behaviour of its individual cells. The coefficient of variation (CV) of ISIs was used to measure irregular firings, while bursting activity was measured by means of the asynchrony index (AI), the ratio of the mode to the mean ISI [45]. Small values of AI<1 indicate a large portion of short ISIs compared to the mean firing rate.

**Fig 3:**
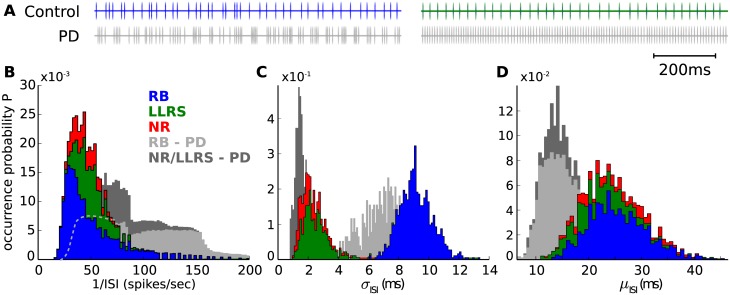
Firing patterns of the three types of STN neurons. **A**: Two recorded examples of STN neurons in a phasic channel show irregular (left) and regular (right) firing patterns, as well as the behaviour of the same neurons during the Parkinsonian ‘off’ state. In this example, the RB neuron exhibits rhythmic bursts in the beta band. **B**–**D**: ISI distributions of STN neuron types, superimposed on stacked histograms. The letters *μ* and *σ* represent the mean and standard deviation repsectively, across all spike events of each neuron. The gray shadow (when both shades are combined) represents the total STN distribution during the ‘off’ state, while the light gray shadow shows only the distribution of RB neurons.

Fig 3 illustrates that, indeed, excessive beta activity observed in the Parkinsonian ‘off’ state is orchestrated by rhythmic bursts, produced by the rebound-bursting (RB) STN neurons. It is worth noted that the term “rebound” here is used only as a part of the name of this neuron type since, in this case, RB neurons produced bursts without the presence of inhibition.

Although the rest of the neurons in the STN exhibited highly regular behaviour that did not change between ‘on’ and ‘off’ states (CV = 8.9% ± 0.02, AI≈ 1 ± 0.05), the firing patterns of RB neurons were less regular (on: CV = 37% ± 0.08, off: CV = 50.2% ± 0.08), and produced rhythmic bursts (on: AI = 0.89 ± 0.3, off: AI = 0.69 ± 0.2) while in the ‘off’ state (for an example see Fig 3.A). Hence, by combining bursts with ISIs approximately between 5 and 10 ms (Fig 3.B) interrupted by longer ISIs between 13 and 40 ms, this rhythmic activity of RB neurons can explain the frequency peak at 20Hz that was illustrated in Fig 2.E. The statistics presented in this paragraph concern all neurons in a phasic STN channel, averaged over all simulated trials. The tonic channels of the same simulations were not taken into account here as the inhibition of the tonic GPe introduces an extra level of complexity to the analysis of the STN patterns.

Furthermore, we observed that tonic activation of GPe was able to (weakly) increase synchronization between STN neurons for approximately 400 ms, following the stimulation of the underlying BG channel. Fig 2.D shows that this synchronization in phasic STN channels was influenced by dopamine, in an inversely-proportional manner. This behaviour is not surprising, since the lack of dopamine was shown to cause increases in the same frequency bands both in GPe and STN, thus facilitating synchronization. In addition, the overall low levels of cross-correlation are also expected, as the metric *C*_*xy*_ in Fig 2.D reflects an average comparison between all (largely uncoupled) simulated neurons in the STN. To further confirm this observation, we created a number of surrogate time series of the binned spike events of each neuron, randomly shuffled over time [46], which destroyed any linear correlations between spikes. A comparison with the original time series produced by our model showed that there was close-to-zero correlation between synchronization and dopamine in the case of the surrogate data, in contrast to the former case, thus the null hypothesis of uncorrelated noise can be rejected.

Finally, although STN neuron synchronization decays over time in our simulations, it is possible that in biological BG this effect would be echoed back via increased beta oscillations in the BG-thalamo-cortical loop. To test if the STN syncronous state can be maintained when it is driven by extrinsic beta activity, we compared the STN behaviour of the above example, as opposed to the case when the simulated cortical ensembles oscillate at a upper-beta frequency (*f* = 25Hz) with a weak amplitude of *A* = 6 spikes/sec, without changing the overall cortical firing rate. As a measure of synchrony, we extracted the instantaneous phases of each STN neuron using the Hilbert transform across each mean-centred and Gaussian-smoothed spike train. The synchrony Φ was then calculated as the average of $\frac{1}{N}\sum_{j}^{N}e^{i\theta_{j}^{H}\left(t\right)}$ over time *t*, where *N* is the number of STN neurons and $\theta_{j}^{H}\left(t\right)$ represents the instantaneous Hilbert phase of the neuron j. This method was selected for its tolerance to amplitude changes [47], since cortical oscillations at the same frequency can increase the amplitude of the STN emerging beta. After 100 simulations for different initial conditions, the static case resulted in average synchrony Φ_*ph*_ = 0.26 ± 0.01 in a phasic microscopic channel and Φ_*to*_ = 0.32 ± 0.01 in a tonic channel, where the symbol ± represents standard error. This 23% higher value in the tonic case was anticipated as the GPe is active and able to provide inhibitory feedback to the STN. On the other hand, cortical beta oscillations raised the syncrony in phasic channels to Φ_*to*_ levels ($\Phi_{ph}^{25Hz}=0.34\pm 0.02$), even though the GPe remained silent, therefore supporting the initial hypothesis that STN synchronization could be maintained if it could cause excessive beta to the cortical areas that project back to the STN.

### Parkinsonian beta activity can be locally-generated but cortically-entrained

Within the BG, recordings of PD patients and primate subjects show exaggerated beta oscillations in the STN [11, 28, 48, 49] that correlate with the pathological symptoms of PD [50, 51] and exhibit high local coherence [52]. Although a well studied phenomenon, the literature provides conflicting evidence regarding the source of these oscillations, which are thought to either be generated internally, via the STN-GPe reciprocal coupling [21, 36, 53, 54], or within other BG nuclei, such as the striatum [55], or in certain areas of the cerebral cortex [10, 49].

One compelling hypothesis, presented in [11], is that upper-beta oscillations of the motor cortex entrain beta activity generated within the BG, which however peaks in the lower-beta band, during the Parkinsonian ‘off’ medication state. Our results support this hypothesis and provide a potential explanation that points to the internal dynamics of the STN rebound bursting (RB) neurons as the source of these pathological oscillations.

In the simulated Parkinsonian state of a phasic channel in Fig 2, our model indeed produced excessive lower-beta oscillations, enhanced by both dopamine depletion and the potentiated cortico-subthalamic projections. Despite its influence on beta amplitude, however, the lack of dopamine was not sufficient to increase the average synchronization between pairs of STN neurons, as it is found *in-vivo* [52], unless the STN activity was measured transiently, right after the halt of cortical beta oscillations (Fig 2.D). To solve this problem and achieve a synchronous steady state, the model was stimulated with a weak oscillatory cortical input in upper-beta band (25 Hz), which was found able to entrain the STN neurons and increase the average instantaneous phase-synchronization Φ by 31%. The oscillatory behaviour that emerged after this modification closely resembles STN field potential recordings in the motor BG of PD patients in [41], and reveals a difference between the role of lower and upper beta bands, which is consistent with the discussion in [11].

### Only low cortical frequencies can be maintained throughout the BG structure

When the BG model received oscillatory input from the simulated cortical ensembles, it exhibited a mixed behaviour. In this experiment, a *phasic* BG channel was stimulated by a cortical ensemble with frequency *f*_1_ ∈ (0, 80) Hz and amplitude *A*_1_ = 10 spikes/sec, while a second neighbouring channel received input from a *tonic* ensemble with amplitude *A*_1_ = 3 spikes/sec, frequency *f*_2_ = *f*_1_ and random relative phase *ϕ*_2_ ∈ [0, 2*π*). The aim here was to explore the ability of the model’s internal dynamics to filter out some frequency bands while preserving others, which would allow the discrimination between cortical frequencies that pass to the thalamus and end up back in the cortex. The metrics used for this analysis were the power of the examined frequency band in each nucleus and the coherence between the cortical inputs and the nuclei. Frequency spectra were calculated using the same methodology as before, while coherence was defined as the normalized cross-spectral density between the above sources.

**Fig 4:**
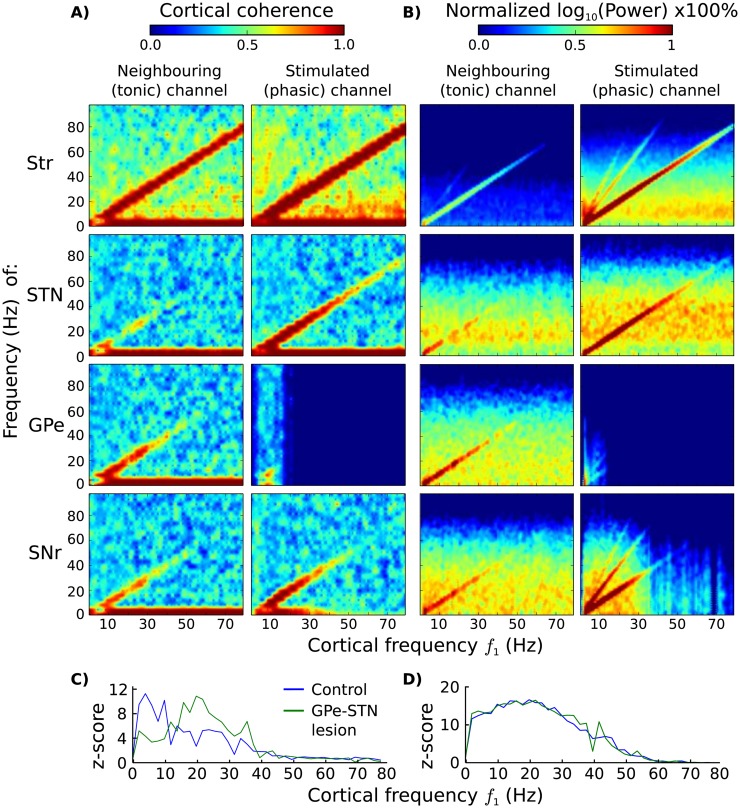
Cortical coherence throughout the BG. **A**) Cortical coherence and **B**) frequency spectrum and of the BG nuclei for different cortical frequencies *f*_1_. A unified spectrum for all striatal populations was included (Str), since they all exhibited very similar behaviour. **C**, **D**) Z-score transformation of the cortical frequency *f*_1_ in the STN power spectrum of the neighbouring (C) and the phasic (D) channels.

In low frequencies, between 0 and 30 Hz, the oscillatory patterns of the cortical inputs were largely replicated in all BG nuclei, in both the phasic and neighbouring tonic channels, with the exception of the tonic STN. In contrast, cortical activity at higher frequencies was preserved in the striatum but declined in subsequent structures. This is evident in Fig 4.A and 4.B, where frequency power and coherence match at most input frequencies. One clear reason for this decline is the blockage of the GPe and SNr activity in the phasic channel, that occurred at high frequencies due to the strong striatal inhibition. However, the fact that cortical oscillations in certain frequency bands could not be followed by the tonic STN (see low beta and gamma bands), or by the tonic GPe and SNr that were not silent (see gamma band), points to the existence of another mechanism that filters out certain frequency bands.

One candidate explanation for the filtering of certain frequencies by the tonic BG, which was revealed here, regards the inter-channel competition that was evoked by the MSN collaterals and the multi-channel excitation from the STN. In particular, neurons of the STN that correspond to the stimulated (phasic) channel, were able to send EPSPs to GPe neurons of neighbouring channels, which in turn inhibited neighbouring STN neurons and cancelled out the initial oscillatory EPSPs from the cortex. Fig 4.C shows that, without the influence of GPe inhibition, tonic STN neurons (with phasic neighbours) tended to adapt to cortical oscillations at frequencies 13–40 Hz with a maximum effectiveness, while under normal conditions, this frequency band shifted to 0–12 Hz. Phasic channels were not influenced by GPe inhibition (Fig 4.D). In a similar fashion, this self-cancelling mechanism affected the entire BG circuitry via STN EPSPs, and facilitated the blockage of high-frequency cortical coherence.

Furthermore, the striatum produced harmonic oscillations (mainly in MSN_*D*1_ neurons), at frequencies limited to the low and gamma ranges (Fig 4.B). Unlike cortical oscillations, harmonics passed only to the SNr of the phasic channel via the direct pathway, and they were strongly amplified along this route. This effect of cortical harmonics could constitute a BG mechanism that facilitates inhibition over excitation, and allows inhibitory BG pathways to be more tolerant to different phases than the hyper-direct pathway.

Finally, we ran the same experiments in PD conditions to evaluate its impact on the above mechanisms. The only noticeable effect was the increase of the frequency range of cortical oscillations that can be maintained throughout the BG. The maximum frequencies increased by 20%–40% across all BG nuclei. This result was consistent for both frequency power and coherence.

### Cortical frequency defines the effective connectivity of the BG pathways

The effective connectivity between the BG structures over a certain period of time can be measured by calculating the causal interactions between their corresponding spiking time series, using a variety of statistical methods. In this work, we used pairwise transfer entropy (TE) [56], a generalization of granger causality, when the Gaussianity of the time series cannot be assumed [57]. TE between two time series *X* and *Y* at time *t* measures to what extent the couple (*X*_*t*−*τ*_, *Y*_*t*−*τ*_) is more resourceful in forecasting *Y*_*t*_, than just the value of *Y*_*t*−*τ*_ [58]. It is expressed as

$$\begin{array}{c} T_{X\to Y}=-\sum_{t}p\left(x_{t},x_{t-\tau }^{\left(k\right)},y_{t-\tau }^{\left(l\right)}\right)\log\frac{p(x_{t}|x_{t-\tau }^{\left(k\right)},y_{t-\tau }^{\left(l\right)})}{p(x_{t}|x_{t-\tau }^{\left(k\right)})} \end{array}$$ (1)

where *k*, *l* are the lengths of the events *x*_*i*_ ∈ *X* and *y*_*i*_ ∈ *Y* respectively, and the time constant *τ* indicates the interval between the two measurements, i.e. the time delay of the information flow. The choice of *τ* in measuring TE between neuronal ensembles is very important and can lead to significantly different numbers (see S2 Fig), that might be influenced by the delays of different afferent connections. A reasonable choice, which was also adopted in this work, is to calculate the TE that arises on the timescale of the AP propagation via the chemical synapses between the examined ensembles.

For the generation of the time series, 10 seconds worth of data was recorded, for every frequency of cortical oscillation between 1 and 100 Hz. The amplitude of the oscillation in the examined BG channel was set to *A* = 10 spikes/sec while oscillations in neighbour channels were limited to 3 spikes/sec. The phase offset *ϕ* between cortical oscillations of this channel and other neighbours was randomized uniformly in every run. Finally, the spiking activity of each BG nucleus was summed for each millisecond and then low-passed using a discrete-time RC filter (*RC* = 2, *dt* = 0.1). For the calculation of the probability density functions in Eq 1, Kraskov’s kernel estimator was employed, a non-parametric method without the need for fine-tuning that is proven to be suitable for our perpose [59].

**Fig 5:**
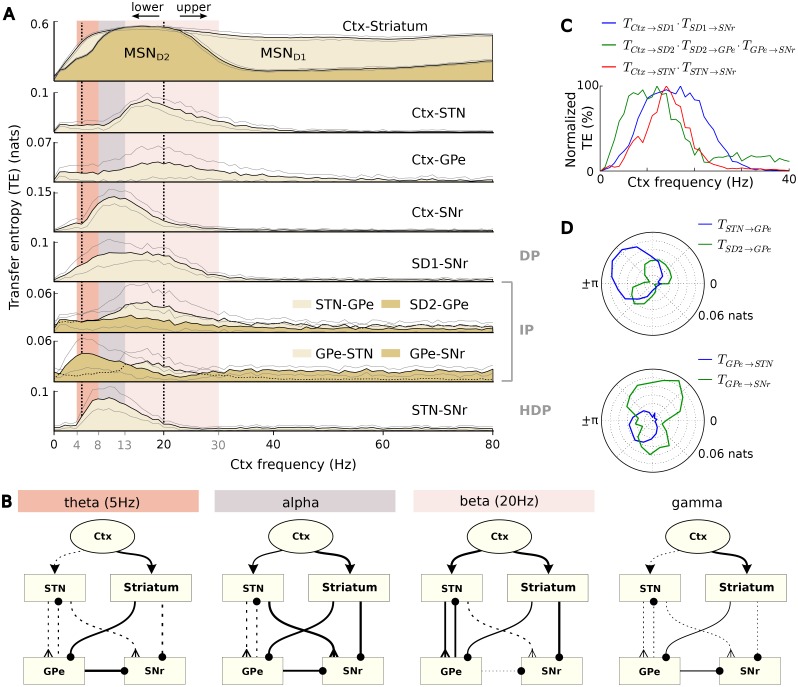
Effective connectivity of the BG model. **A**: Spectrum of TE for the connections of the BG circuit. DP, IP and HDP stand for the direct, indirect and hyper-direct pathways respectively. The plotted black and gray lines represent mean and standard deviation respectively, calculated over 100 10-second simulations for each value of cortical frequency. The vertical black dashed lines highlight the exact frequencies that were used in (B). **B**: Resulting effective connectivity of the BG for different cortical frequency ranges. For the theta and beta bands, only the representative frequencies 5Hz and 20Hz were used respectively, as the system exhibited different behaviours across these two ranges. Thickness of synaptic connections represents TE (normalized across the frequency spectrum) and the solid lines show the dominating pathways. **C**: Activation of the three main BG pathways for different cortical frequencies. **D**: TE of GPe afferents versus efferents, for different phase offsets *ϕ* and alpha cortical rhythms. When 0 ≤ *ϕ* ≤ *π*, oscillation peaks in the phasic cortical channel precede in time oscillation peaks in the tonic channel.

Fig 5.A illustrates the resulting spectrum of TEs between the cortex and the BG nuclei (first half) and for the main pathways of the BG circuit (second half). We observed a clear distinction between input frequency bands, giving rise to completely different behaviour in the model (Fig 5.B). The greatest variation arose in low-frequency bands, between 4 and 30Hz, under the very conditions that are necessary to allow the relay of information via the BG.

If viewed as information channels, the three major BG pathways remained widely open during stimulation at alpha frequencies (Fig 5.A and 5.B gray column). In the indirect pathway, striatal neurons were able to affect the behaviour of the SNr via the GPe, bypassing modulation by the STN-GPe loop and, as a result, the input-output information flow in the BG maximized compared to any other frequency band (Ctx-SNr in Fig 5.A). In the case of the lower-beta band, greater information flow from the cortex allowed the STN to modulate the indirect pathway, and to maintain a higher impact than the GPe on the SNr. Stimulation at upper-beta frequencies exposed a different balance, where the flow of information via the STN and GPe is restricted to interactions within the STN-GPe loop, and thus the SNr behaviour is dictated by the MSN_*D*1_ inhibition. At gamma frequencies, a cortical information blockade turned the STN into a local-circuit component that affected the SNr only via GPe inhibition. The full indirect pathway dominated the BG behaviour and blocked cortical information flow.

Finally, below alpha, the impact of the GPe on the SNr was maximal at theta frequencies (4–8Hz), even though the information flow from excitatory sources towards the GPe abated considerably. In fact, the amount of *T*_*GPe*−*SNr*_ was found to have increased by 84 ± 58% compared to the sum of TE towards the GPe, a fact that leads to the hypothesis that, under these conditions, some of the information that arrives to the SNr is generated within the GPe.

Fig 5.C summarizes the above observations and illustrates the impact of the cortical frequencies on the activation of the three main BG pathways. This analysis was based on a heuristic method, where the values of TE between the consecutively connected nodes of a pathway were multiplied and then normalized with respect to their distribution across cortical frequencies. Interestingly, as evidenced by this figure, different frequency bands give rise to different combinations of open pathways, increasing the repertoire of potential functions that the BG are able to perform.

Furthermore, we observed that in certain low frequencies, the phase offset *ϕ* between the two oscillating cortical ensembles was able to change how the STN and GPe interact with their adjacent nuclei. In Fig 5.D, different phase offsets between alpha oscillations were able to block, or reverse the direction of information flow between STN and GPe, which was also accompanied by a pronounced effect on the pathways that include them. This was more evident when the strong cortical signal of the phasic channel (10 spikes/sec) preceded in time the weaker (tonic) oscillatory signal of a neighbouring channel, i.e. 0 ≤ *ϕ* ≤ *π*. In this case, the flow of information was stronger towards the STN, and the activation of the hyper-direct pathway was largely modulated by the influence of GPe (Spearman’s correlation between *T*_*STN*−*GPe*_ and *T*_*STN*−*SNr*_: *ρ* = −0.72, *p* ≈ 5 × 10^−17^), while in the opposite case, when −*π* ≤ *ϕ* ≤ 0, the prevailing direction of the flow also reversed. This effect had significant ramifications for the balance between hyper-direct and indirect pathways which was found to be strongly correlated with the direction of flow between the STN and GPe (Spearman’s correlation between $\frac{T_{STN-GPe}}{T_{GPe-STN}}$ and $\frac{T_{STN-SNr}}{T_{GPe-SNr}}$: *ρ* = −0.44, *p* ≈ 3 × 10^−05^). This set of observations provides insight into the modulation mechanism of the STN-GPe loop and indicates the importance of phase-to-phase coherence in low-frequencies.

As previously, we used scrambled surrogate testing to confirm that our observations were not a result of uncorrelated noise. After randomly shuffling the time-series of each nucleus for 1000 times, the average TE for all connections became 2.6 × 10^−3^ ± 8.5 × 10^−4^, and it was similarly distributed across different frequencies. Hence, as these values are considerably lower than the resulting TEs in Fig 5 and the dependency of the input frequecy was destroyed, the null hypotheses of (1) no significant information transfer between source and target nuclei and (2) absence of correlation between frequency and the measured TE can be both rejected in the above observations.

#### The effect of the relative phase

As illustrated in Fig 4.C, the existence of a closed loop between STN and GPe contributes to the maintenance of cortical frequencies in the alpha band, and their blockage in higher bands. Without feedback inhibition, the rhythmic bursts of STN-RB neurons succumb to the cortical beta rhythms, due to their natural tendency to engage in beta activity (Fig 3). Then, we showed that at the same alpha frequencies, the direction of information flow inside the STN-GPe loop changes depending on the relative phase of the stimulus versus other background oscillatory activity that influences neighbouring areas. Both these remarks highlight the strong functional connection between this internal loop and cortical low oscillations.

**Fig 6:**
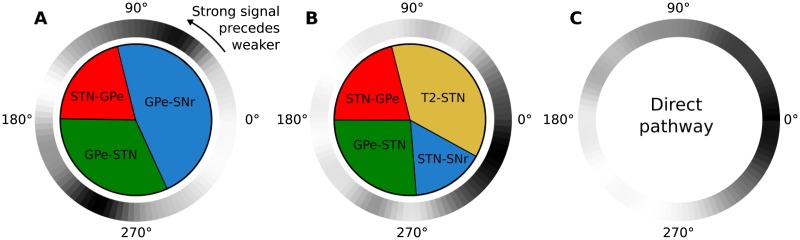
Competition of STN-/GPe-mediated pathways triggered by cortical alpha. Inner circle: Synaptic connection with the highest TE in the GPe (**A**) or the STN (**B**), for different phase offsets *ϕ*. Outer circle: Normalized TE across the values of *ϕ* in the indirect (**A**), hyper-direct (**B**) and direct (**C**) pathways as defined in Fig 5. Black and white colours correspond to 100% and 0% respectively.

A closer examination of the effect of the phase offset *ϕ* reveals a number of modes of the STN-GPe function, able to trigger a competition between the two involved pathways (indirect/hyper-direct) over the range of possible values of *ϕ*. An example behaviour for alpha frequencies is illustrated in Fig 6, where both the absolute magnitude and the sign of *ϕ* ∈ [−*π*, *π*) contribute to the outcome of this competition. (See S3 Fig for more frequency ranges with similar behaviour.) While large alpha offsets always activate the indirect and suppress the hyper-direct pathway, values close to zero have the opposite result, notably when the strong input signal is preceded by background oscillation in neighbouring channels. This asymmetry cannot be observed in the direct pathway, which is not directly influenced by either STN or GPe. Thus, its TE maximises monotonically and smoothly around *ϕ* = 0.

## Discussion

The first main contribution of this work is a new detailed neural model of the BG canonical circuit which can be used as a tool for both producing and testing hypotheses related to the BG function. Due to the enormous interest in this brain region, there are numerous available computational modelling approaches in the literature [19–25, 60] (for reviews see: [26, 27]). A series of modelling research has utilized the conductance properties of STN and GPe neurons in order to explore synaptic and cellular mechanisms of neural oscillations in the BG. Some of these approaches aim to capture the exact electrophysiology of BG nuclei [60, 61] while others focus on the mathematical interpretation of conductance properties [62]. However, whereas conductance-based models provide a much greater level of detail than simple phenomenologial neuron equations, this advantage is, to some extent, lost in large-scale simulations. Their large number of parameters increases the difficulty of achieving biologically plausible variability and their much higher computational cost limits the number of units that can be used in a simulation. The current study is focused on features that have a clear and strong effect in BG network dynamics, such as short-term plasticity, number of neurons (and ratios of types of neurons) in each nuclei, both electrical and chemical synapses and spatially embedded striatal circuitry. To our knowledge, has so far been no large-scale neural model of the complete BG circuitry, that integrates this number of features of the BG physiology.

Oscillatory behaviour emerged through the model dynamics that resembles various known BG phenomena. Although similar oscillatory frequencies have been observed in a number of other early bottom-up studies, including [19] and [63], the fact that the tuning process followed here was based only on simple firing rate rules and neuron electrophysiology, updating the maximum synaptic conductances and the internal phenomenological parameters of the individual neurons respectively, gives extra value to the presented novel results. As our simulations show, the frequency of the cortical input can be maintained throughout the BG structures and dramatically changes the way that the BG circuit operates.

One of the weak points of low-level computational models is that the large number of parameters entailed can generate broad types of behaviour, depending on the tuning applied. In our approach, we tried to minimize this effect by tuning this model from the bottom up, almost entirely on electrophysiological studies while the focused level of behaviour is oscillations of neural populations. In the rest of this section, we discuss the consistency between our results and previously published experimental data and theories, and we provide a number of predictions that are supported by our simulations and can be tested in further experimental or modelling work.

### Is the source of parkinsonian beta a combination of STN dynamics and cortico-subthalamic entrainment?

Numerous associations can be made between the oscillatory behaviour of our model and experimental data both in the healthy and Parkinsonian BG. In [4], Leventhal et al. discovered that beta power in the cortex and the BG of healthy mice changes distinctively during behaviour. They also measured coherence and correlation of frequency bands throughout the BG and found that, during their behavioural experiments, coherence was maintained at both alpha and beta frequencies but disappeared at higher frequencies. In Fig 4.A. we observed the same phenomenon in our modelled BG both in a stimulated channel that was driven by a phasic, oscillatory cortical activity, as well as in neighbouring areas.

The clear leading role of low cortical oscillations in affecting the BG function shown here is also supported by EEG studies with PD patients. Ahn et al. found correlations between various cortical areas and single STN units in beta band and not in gamma [64]. In addition, Shimamoto et al. found excessive synchronization between local field potentials in M1 and STN units, at theta, alpha and beta frequencies [65]. However, more complicated oscillatory phenomena found in the latter study, such as phase amplitude coupling, that also facilitates gamma synchrony, were not replicated in our experiments, as the simulated cortical activity was always set to follow a single-frequency oscillation. Finally, Ahn et al. [66] investiageted this topic using a small-scale computational model and proposed that excessive Parkinsonian beta oscillations could be due to both the cortical and BG mechanisms.

Within the microcircuit, the current work predicts that excessive beta activity is generated locally, by the dynamics of a sub-type of STN neurons, but entrained by cortical activity at a slightly higher frequency. Nevertheless, the question remains of how cortical upper-beta can be the source of this entrainment. More light can be shed at the single-unit level, where the majority of the STN neurons showed a mixed rhythmic bursting behaviour, similar to recordings in [44], with a frequency peak at around 18 Hz (Fig 3.A). In particular, we observed that, without the influence of the GPe, which is locally inhibited on phasic microscopic channels, and with excessive excitation from the cortex, the rebound-bursting STN neurons generate free and uncoupled oscillations, resulting mainly from their internal dynamics. Since they are uncoupled, these oscillations are prone to entrainment by external stimuli, insofar as those stimuli also oscillate at a compatible frequency, such as in the experiment described above.

The plausibility of the firing patterns of both the pathological and healthy simulated STN neurons can be supported by a number of empirical studies. The positively skewed distribution of the inverse ISIs of these neurons, shown in Fig 3.B, agrees well with the distribution of single neuron firing rates, recorded in the STN of healthy monkeys [43]. After a treatment with the neurotoxin MPTP, which is known to cause Parkinsonian-like symptoms [67], the distribution of firing rates shifted towards higher values and had a flattened profile, a feature that was also captured by the simulated Parkinsonian ‘off’ state and illustrated in the same figure. Furthermore, the ratio of burst-like neurons and the distribution of mean ISIs for each STN neuron in Fig 3.D is consistent with the results of multi-electrode recordings in human PD patients in [44], where the power spectra of individual STN neurons were found to peak at 17.9 ± 6 Hz.

The pathological mechanism we propose here could be further investigated experimentally, with a signal-cancellation technique either at the level of the cortex (as in [68]) or directly in the STN using, for instance, DBS electrodes. Our hypothesis predicts that, in highly active areas, a reduction of the influence of cortical upper-beta activity to STN neurons will also reduce the correlation between their spike trains, as they will lose their main source of entrainment, but it will leave the amplitude of lower-beta almost intact.

Moreover, the behaviour of the system in the tonic state reveals the role of the GPe in the generation and maintenance of synchrony within the STN. In Fig 2.B, oscillations in STN and GPe are highly coherent at lower-beta frequencies, a relation that is inversely proportional to the amount of dopamine in the system. In the resting Parkinsonian state, characterized by zero dopamine, enhanced cortico-subthalamic connections and tonic cortical activation, inhibitory feedback from the GPe was able to increase the average instantaneous synchrony Φ of STN neurons by 23% and maintain it for 400 ms after the silence of GPe. This leads to further predictions regarding the interaction between the STN and GPe. First, in periods when the BG input nuclei have areas that are highly active, a subgroup of GPe neurons is expected to be silent, due to high inhibition from MSN_*D*2_ neurons (Fig 1). These periods of silence have been observed before in the GPe [69], and have been linked to striatal inhibition [70], but based on our model, they should also exhibit high correlation with STN activation. Following this vein, long periods of silence in GPe neurons lead to a halt of the only source of inhibitory feedback to the connected STN neurons. As a result, if cortical beta is cancelled out as proposed in the *Results* section, highly active STN neurons are expected to become unable to maintain any synchronous state, and have minimum correlation (as in Fig 2.D), if the duration of this activity exceeds a time threshold.

Apart from the peaks in beta band of the STN power spectrum, Lopez et al. in [41] found a second area, at very high frequencies around 350 Hz, that was evidently high. This activity was shifted towards lower frequencies (250 Hz) without medication for the Parkinsonian symptoms. Although neither case has been captured by our simulations, this was possibly due to the nature of the multitaper method used for spectral analysis, which is insensitive to weak signals at high frequencies [39].

Finally, one more factor that might contribute to the synchronous activity within the STN is the complete BG-thalamo-cortical loop, which involves the hyper-direct BG pathway. Since the STN neurons are able to generate beta patterns spontaneously, they might also be able to to enhance beta activity throughout this loop, even in the case that GPe neurons are locally silent. This can be tested in future work, with an extended version of our model, that also incorporates neural populations corresponding to both thalamic and cortical areas.

### Oscillations and the BG function

Beta oscillations are also prevalent in the healthy function of the BG and they are strongly associated with the motor system of the brain [4, 11, 71–73]. As in the Parkinsonian ‘off’ state [74], they show peaks in both lower and upper-beta ranges, but with a higher median frequency [73], since lower-beta is more sensitive to suppression by dopamine [11]. This feature was reproduced in our simulations, where dopamine was able to control the level of internally-generated lower-beta and effectively reduce it in exchange for upper-beta oscillations, in an almost linear manner (see Fig 2.C). If this ability to change the peak of beta activity is confirmed experimentally, then small fluctuations in rebound-beta that are usually present after the execution of a task [75] or after artificial modulation of dopamine [76] could be reflected in the level of dopamine that changes due to a post-decision evaluation [77].

With regard to their function, one theory proposes that beta oscillations are used to “signal the status quo” across brain regions [72], both at the perceptual-cognitive and motor level, in case that its maintenance is anticipated or intended. Furthermore, a behavioural study with simultaneous, multiple recordings in healthy rats provides evidence that beta oscillations in the BG are strongly related to cue utilization [4], and suggests that high beta activity reflects “a post-decision stabilized state of cortical-BG networks, which normally reduces interference from alternative potential actions”. These views can explain the rigidity and hypokinesia of PD patients who also exhibit abnormally exaggerated beta activity, the observed beta desynchronization in movement preparation and execution [15, 78, 79], as well as the beta rebound in NO-GO decisions [75]. However, it is still unclear why these oscillations have such a strong effect in maintaining the current state of the brain. One recent review suggests that beta oscillations regulate the information capacity of the phasic channels of the loops involving the BG [51].

Here we propose that the BG is able to selectively gate information flow in these channels, via a combination of internally-generated and cortically-driven beta activities, driven by the current level of dopamine and the cortical frequency respectively. We show that, even when their amplitude is kept fixed, different cortical beta frequencies are able to completely change the information flow throughout the BG. The increased flow in low bands in Fig 5 is consistent with the view in [51], and provides a lower bound for the information capacity during the beta regime. More specifically, towards lower beta frequencies, the communication channels of the three major BG pathways open gradually and monotonically, with the same rate but different offsets (Fig 5.C). At 13 Hz, the lowest beta frequency, all three pathways have a global peak, while at the highest beta (30 Hz), they are fully blocked. Hence, the frequency of beta can be used by the cortex as a lever that adjusts the impact of the three BG pathways, and thus plays a decisive role in the generation of movement [13, 15, 80].

Apart from beta, other frequency bands also showed unique characteristics in our simulations. Alpha rhythms resulted in BG effective connectivity changes that were similar to beta, promoting all three BG pathways but with an emphasis on the indirect pathway, and with even higher input-output information flow. In experimental studies, alpha activity has been also very closely associated with beta, exhibiting desynchronization prior to movement and suppression during movement execution [11, 81, 82]. However, these rhythms are considered to have a distinct function [10, 52, 79, 81, 82] and they have been mainly associated with emotional stimuli [83], as well as the attentional system of the brain [10, 11, 84]. In particular, there is cumulative evidence that strong alpha power is able to inhibit task-irrelevant regions in the cortex and thus control information flow [3, 84, 85], while it is argued that alpha desynchronization allows the formation and retrieval of new memories [7]. Finally, alpha power cannot be significantly regulated by the level of dopamine [86], a fact that shows another major difference in the function of these rhythms at the level of the BG. The constant tendency of alpha to promote information flow via the indirect pathway, as observed in Fig 5, agrees well with the above theories. This pathway has been shown to play a critical role in proactive inhibitory control [15, 87] and cause movement suppression [12, 14, 88] by evoking a rapid disinhibition of a subset of SNr neurons. Thus, it is likely that a local increase in alpha power brings the affected cortical region to a stable state, where the cortico-BG-thalamic loop is active but, at the same time, restricted from accessing memory processes and with the corresponding motor responses inhibited.

The two final frequency bands under consideration are theta and gamma. The coexistence of these two bands is a well studied phenomenon in the cortex [89], which, as opposed to alpha and beta, promotes the formation and retrieval of episodic memories via phase-amplitude entrainment between different regions [90]. However, in this study we assess theta and gamma separately to maintain consistency in our methodology and enable the direct comparison with other frequency bands. Cortical theta (∼5 Hz) is involved in various cognitive processes [91] such as memory retention, novelty detection, processing of negative rewards [92] and goal maintenance [93]. Within the BG, theta is found to increase in the rat striatum during a decision-making task [94], while in humans, theta in STN increases during sensorimotor conflicts [95]. Gamma, on the other hand, is mainly associated with active information processing and feature binding [2, 5, 6]. Unlike alpha and beta, it is characterized by high amplitudes during movement [68, 79] and in combination with theta, it facilitates communication between different cortical areas, thus enabling high-level cognitive control such as the simultaneous maintenance of behavioural goals [93].

Interestingly, both gamma band and theta at 5 Hz minimized input-output information flow from the simulated cortical ensembles to the SNr and enabled only the indirect pathway without any modulation from the STN-GPe loop. This similar connectivity pattern indicates that any combination of these two rhythms, as in the aforementioned studies, will also bring the BG to the same state. Hence, our model suggests that cortical information which has been generated and processed via alpha/gamma rhythms is not able to circulate through the cortico-BG-thalamo-cortical loop, without the presence of another low-frequency band.

Furthermore, in the case of gamma, the D1 striatonigral MSNs acted as an information *sink*, receiving strong inputs from the cortex but with a minimal impact on the SNr, while theta rhythms caused GPe to fire spontaneously and dominate the behaviour of the output SNr, thus acting as an information *source*. This effect in the GPe was sensitive to the phase of theta, and it was most prominent when the phase of the phasic channel followed in time the phase of neighbouring-channel oscillations, particularly at an offset of $\phi \approx -\frac{\pi }{2}$ (see S3 Fig).

All things considered, a picture emerges regarding the function of the BG during cognitive processing at theta/gamma rhythms. Our model’s behaviour in these two bands can be viewed as a mechanism that isolates the cortex from the environment, while new information is being processed in multiple cortical regions. In the case of a sensorimotor conflict, theta is increased in the cortex, and the GPe is ‘instructed’ to inhibit SNr in order for the conflict to be resolved. This behaviour is different than in the case of alpha, which boosted the circulation of information via the BG, while inhibiting relevant motor actions with the facilitation of the indirect pathway. Hence, due to the distinction between the aforementioned bands, the cortex acquires the ability to process information through a variety of streams, either by using intermediary subcortical structures, or directly, across different regions.

Although there is no direct connection between GPe and theta function, inhibition of this structure via deep brain stimulation (DBS) has been found to improve cognitive symptoms of Huntington’s disease [96–98], a condition that is associated with episodic memory loss [99] and increased ectopic theta [100] (for a review see [101]), among other symptoms. However, further work is required to verify the above computational predictions, and to answer to the emerging questions regarding the BG function. From an experimental perspective, the role of theta in the GPe, as well as BG effective connectivity changes during behaviour, require extensive investigation. In addition, computational modelling could shed light on the possible combinations of the above mechanisms and the transient versus steady-state dynamics that emerge. Finally, an interdisciplinary investigation on how the effects of the above pathological frequencies can be cancelled out could potentially boost current research on adaptive DBS techniques [102].

### The STN-GPe circuit

The fact that the GPe becomes silent during the phasic mode in our simulations does not contradict with the literature. First, this behaviour reflects to only a very small portion of GPe neurons that are associated with the microscopic channel and exhibits a phasic response. Second, the recordings in our results were conducted for two simulated seconds during which, the cortical input maintained a steady firing rate (either oscillatory or completely fixed). In real conditions, feedback from the BG via the thalamus, would cause changes to the cortex after some milliseconds of the initial GPe inhibition and the input that the BG receives would be modified accordingly. In support of this behaviour, it has been shown in primate recordings [103, 104] that GPe neurons are inhibited transiently for approximately 25 ms after cortical stimulation.

The diversity of the STN-GPe interactions for different cortical phase alignments leads to a hypothesis that breaks this loop down into two coexisting mechanisms. First, the rhythmic inhibition and excitation of the SNr by these two structures may act as a force that attempts to align the phases of different cortical low-frequency signals, in order to achieve optimal communication [5, 6]. However, although perfect phase alignment can maximize information exchange in neural populations, optimal behavioural performance often requires more metastable dynamics [105]. Hence, as an additional mechanism, the BG may be able to impose a veto on two conflicting signals, via the excessive activation of the indirect pathway, in case that the above process results in the wrong alignment, i.e. an amplitude difference that favours the leading signal. This veto can be released if the balance of amplitudes changes, and the leading signal increases its impact on its counterpart. This mechanism could allow the BG to function in a Hebbian fashion and provide the right temporal conditions for the integration of anatomically distinct signals.

The credibility of this hypothesis can be further tested by the addition of neural cortical oscillators as well as a thalamic nucleus to the model presented in this study. This would allow the reverberation of the same cortical signal through the BG and reveal the conditions under which a coalition of cortical ensembles can be phase-coupled via the influence of the STN-GPe circuit.

All in all, the great variability of responses observed during our simulations highlights the extensive repertoire of BG functions. These cannot be completely captured by the analysis of this paper, even in the toy case of fixed dopamine and steady cortical inputs with fixed frequencies. Nevertheless, our study showed that oscillatory frequencies and phase alignments could be the means by which the cortex selects between these functions, and led to a number of predictions that can be tested in future work.

## Materials and methods

The predominant part of the methodology presented here is a new spiking neuron model of the complete motor BG circuitry, partly based on well-established models of various features of the BG nuclei, an early version of which can be found in [106]. In particular, the striatum model was partially adopted from [23], the conductance delays between nuclei were taken from [19] and the parameters for short-term plasticity between the BG nuclei from [22]. This section provides justification and a full description of the mathematical models and the rest of the design choices that were made for this simulation, as well as the tuning process that followed. The source code of this spiking neuron model is written in the programming language Python, using the simulation library Brian [107], and can be found at https://github.com/zfountas/basal-ganglia-model.

### Anatomy

#### Canonical circuit

The internal structure of the majority of the BG forms a single canonical circuit (Fig 1.A), massively replicated in different scales. Macroscopically, it is part of a complex set of parallel loops that involve the thalamus, limbic regions and almost all major regions of the cortex including sensory, motor and associative areas [8, 108]. However, at the level of the BG, these loops can be further broken down into parallel microscopic channels that involve the same canonical circuit and, with a small overlap, maintain the anatomical division and somatotopic organization found in the cortex [19, 109, 110].

A widely accepted hypothesis is that these microscopic channels represent different competing “action requests” [33] that originate from the cortex. These requests are processed by the BG circuit, which, under some conditions [106], is able to select the most salient (or urgent) potential action [13, 19, 111].

Along these lines, the model presented here comprises six neural populations that correspond to the four major nuclei of the biological BG and form the canonical circuit described above. These include the striatum and the subthalamic nucleus (STN), the two main input structures in the BG, the external part of the globus pallidus (GPe), as well as the substantia nigra pars reticulata (SNr), one of the two output structures of the BG. Furthermore, the effect of the pars compacta part of the substantia nigra (SNc) is realized through the concentration of the neurotransmitter dopamine (DA) in the different parts of the network (green colour in Fig 1.A).

The internal structure of the striatum has been modelled using three different groups that correspond to its three major neural populations. The first two groups constitute the two categories of medium spiny-projection neurons (MSNs), divided based on the dominant type of their dopamine receptors, which belong either to the D1- or D2-like families. Depending on their category, these neurons are either enhanced (MSN_*D*1_) or depressed (MSN_*D*2_) by the presence of dopamine. They have been predicted to comprise the 99% of the striatal volume [112], a number that was also maintained here.

Finally, the remaining 1% of the striatum is occupied by fast-spiking gabaergic interneurons (FSIs) that are affected by both types of dopamine receptors and are highly interconnected with both electrical and GABAergic synapses. Despite their small concentration, FSIs have a great influence on the rest of the striatum, and it has been shown that inhibition from a single FSI cell is able to block action potentials in large numbers of MSNs [113].

To estimate the number of neurons within each nucleus, we kept the same ratios of neurons found in rat BG [114]. The final numbers can be found in Table 1 and result in a total of 9586 neurons that form the BG network. The probability for a connection between two neurons *P*_*X*−*Y*_ depends on the pre- (*X*) and post-synaptic (*Y*) nuclei and can be found in the same table. The values of these probabilities were inferred by the same method that was used for the random model of striatum in [23]. For connections that involve only striatal neurons, the required data was obtained by the spatially embedded model in [112], while the model in [22] was used for any other connection.

**Table 1:** Network parameters.

Parameter	Source	Parameter	Source
*N*_*MSN*_ = 2790000 × 0.99/*S*	[20, 114]	*P*_*Ctx*−*STN*_ = 0.03	[23, 112]
*N*_*FSI*_ = 2790000 × 0.1/*S*	[20, 114]	*P*_*Ctx*−*MSN*_ = 0.084	[23, 112]
*N*_*GPe*_ = 46000/*S*	[114]	*P*_*Ctx*−*FSI*_ = 0.084	**
*N*_*STN*_ = 13600/*S*	[114]	*P*_*SD*1−*SNr*_ = 0.033	***
*N*_*SNr*_ = 26300/*S*	[114]	*P*_*SD*2−*GPe*_ = 0.033	***
*N*_*T*_*i*__ = 1000	Assumed	*P*_*STN*−*SNr*_ = 0.3	***
*R*_*GPe*_*A*__ = 0.0405	[30, 69]	*P*_*STN*−*GPe*_ = 0.3	***
*R*_*GPe*_*B*__ = 0.85	[69]	*P*_*GPe*−*STN*_ = 0.1	***
*R*_*GPe*_*C*__ = 0.1095	[30, 69]	*P*_*GPe*−*SNr*_ = 0.1066	***
*R*_*RB*_ = 0.6	[115]	*P*_*GPe*−*GPe*_ = 0.1	***
*R*_*LLRS*_ = 0.25	[115]	*P*_*SNr*−*SNr*_ = 0.1	Assumed
*R*_*NR*_ = 0.15	[115]	$P_{MSN-MSN}^{int}=0.0718$	****
*S* = 300	*	$P_{MSN-MSN}^{ext}=0.0082$	****
$P_{FSI-MSN}^{int}=0.2925$	****	$P_{FSI-FSI}^{int}=0.5864$	****
$P_{FSI-MSN}^{ext}=0.0314$	****	$P_{FSI-FSI}^{ext}=0.0092$	****

* Assumed to be adequate for 3 channels

** Same as *P*_*Ctx*−*MSN*_ [112]

*** Calculated keeping the ratios from [22]

**** Calculated using probability distributions from [112]

#### Lateral inhibition

Within each nucleus in our model, there are three largely isolated subgroups that correspond to three microscopic channels of the BG circuit. As mentioned before, the BG preserves the anatomical organization of their cortical inputs, thus connections between nuclei are mainly topographic and influence only the same channel in the target nucleus. As an exception, the STN glutamatergic efferents cause diffuse excitation [111], equally distributed across adjacent channels. In addition, evidence for local axon collaterals in GPe [116] and SNr [117] suggests that lateral inhibition in these structures also spans to neighbour functional subdivisions, and thus, it is also considered diffuse.

The striatum, on the other hand, has more complicated intrinsic connectivity which arises from both its enormous size and the extensively overlapping network of axon collaterals [118, 119]. A large debate has been provoked regarding its connectivity structure and computational function. The “domain” theory [120] suggests that the striatum is divided into groups, or domains, of highly inter-connected neurons that form local winner-takes-all elements, while more recent studies show that striatal lateral connectivity is weak and sparse, and indicate that the striatal computational element should be spread across the MSN network [112, 121].

Here we use two different probability values *P*^*int*^ and *P*^*ext*^ that represent lateral connections within and between striatal channels respectively, thus allowing both views of localized and sparse connectivity to be tested. To calculate the values of these probabilities for each type of striatal local connection we generated a spatial model of two adjacent striatal microscopic channels and calculated the internal and external mean connection probabilities. Assuming that all neurons of a single channel are limited within a spherical boundary, the radius of this sphere can be found from $R={\left(\frac{3V}{4\pi }\right)}^{1/3}$, where $V=\frac{N_{ch}}{84900}mm^{3}$ is the simulated striatal volume (since in 1*mm*^3^ there are 84,900 neurons [112]) and *N*_*ch*_ = (*N*_*MSN*_ + *N*_*FSI*_)/3 is the number of neurons within this sphere. For the values of *N*_*MSN*_ and *N*_*FSI*_ that are given in Table 1, *R* = 205.8*μm*^3^.

The estimated probabilities, which are also shown in the same table, where found after the calculation of the average number of contacts within and between these two adjacent areas, using the distribution of expected number of intersections with respect to the distance between the somas of two neurons, in [112].

In addition, the striatum was shown to be asymmetric with respect to inhibition that MSN_*D*1_ and MSN_*D*2_ neurons receive, both in conductance strength and number of connections. Local MSN collaterals have fewer and weaker connections that arrive to striatopallidal neurons than the opposite [122, 123], while FSIs also target mostly MSN_*D*1_ [124] neurons. This strong inhibition of the direct pathway compensates for the over-excitement of these cells via *D*1 receptor activation, and thus brings more balance to the intrinsic activity of the striatum.

To account for the effect of the above asymmetries, the probabilities in Table 1 change to *P*_*D*2−*D*1_ = *P*_*MSN*−*MSN*_ * *W*, *P*_*D*1−*D*2_ = *P*_*MSN*−*MSN*_ * (2 − *W*), *P*_*FSI*−*D*1_ = *P*_*FSI*−*MSN*_ * *W* and *P*_*FSI*−*D*2_ = *P*_*FSI*−*MSN*_ * (2 − *W*), where *W* defines the trade-off of inhibition between the direct and indirect striatal neurons. The default value used is *W* = 1.5 which is consistent with previous studies [34, 122, 124]. Finally, changes in maximum conductances *G* of collateral MSN connections were inferred from [122]. For recurrent MSN_*D*1_ connections *G* = 1.2 * *G*_*SD*−*SD*_ and for MSN_*D*2_ to MSN_*D*1_ connections *G* = 0.4 * *G*_*SD*−*SD*_.

### Mathematical models

#### Neuron dynamics

The electrical activity of individual cells of the BG was simulated using the single-compartmental “simple model” that was proposed by [29, 125]. In this phenomenological model, the membrane potential *v* of the neuron is governed by the equation

$$\begin{array}{c} C\frac{dv}{dt}=k\left(v-v_{r}\right)\left(v-v_{t}\right)-u+I+CN\left(0,\sigma^{2}\right) \end{array}$$ (2)

where *I* is the dendritic and synaptic current, *C* the membrane capacitance of the cell body, *v*_*r*_ the resting membrane potential, *v*_*t*_ the instantaneous threshold potential, *k* an abstract parameter and *u* is an abstract recovery variable with

$$\begin{array}{c} \frac{du}{dt}=a(b(v-v_{r})-u) \end{array}$$ (3)

In this equation, *a* and *b* are two additional abstract parameters of the model. Finally, the neuron is said to fire a spike when its membrane potential exceeds the threshold value *v*_*peak*_. In this case, the variables of the model reset to general cation currents

$$\begin{array}{c} \begin{array}{c} v\to c\\ u\to u+d \end{array} \end{array}$$ (4)

where *c* and *d* are further abstract parameters.

If tuned properly, this model is able to display the known types of dynamical behaviour of all cortical and sub-cortical neural cells, and to quantitatively reproduce their sub-threshold, spiking, and bursting activity in response to pulses of DC current [125]. In addition, the recovery variable in (3) could be tuned to represent a specific mechanism of an ion channel such as the calcium-activated potassium channels in STN neurons [126] as will be shown in section STN model.

The Eqs 2 and 3 can be reduced to a simpler form, originally presented in [29] and widely used, which contains only two independent parameters. However, the choice of the current extended form is considered more appropriate for this study, since the majority of the parameters and the variables here acquire biophysical meaning, which simplifies the complexity of calculations and tuning. For example, electric potentials, such as *v*, are represented in *mVolts* and the input current *I* in *pAmperes*.

Heterogeneity of the neurons in the network is achieved by the stochastic perturbation of the capacitance *C* of each neuron by a small random factor, sampled from a Gaussian distribution with mean *C*_*μ*_ and standard deviation 0.1 × *C*_*μ*_. In addition, every neuron includes a general Gaussian noise factor $N(0,\sigma^{2})$, added to its membrane potential, with a constant standard deviation (*σ* in Eq 2), which depends on the type of the neuron. This term represents the effect of external afferents that are not part of the this model and are considered stable during our simulations.

#### Synaptic dynamics

Neurons in the network are connected with up to three different categories of synapses, depending on their position and type. A synapse can be either simple chemical, chemical plastic or electrical. The simple case of a static chemical synapse is implemented with a standard conductance-based model [127] with different parameter values for different neurotransmitters and connectivity. At any given point of time *t*, the current of each synapse can be described with

$$\begin{array}{c} I_{ij}^{s}\left(t\right)=\{\begin{array}{cc} G_{ij}e^{-\left(t-\left(t_{i}+\lambda \right)\right)/\tau_{s}}\left(E_{s}-v_{j}\right) & \;\text{if}\;t\geq (t_{i}+\lambda )\\ 0 & \;\text{if}\;t<(t_{i}+\lambda ) \end{array} \end{array}$$ (5)

where *t*_*i*_ is the time of last firing of neuron *i*, λ is the delay of the synapse, *G*_*ij*_ is the maximum conductance of the synapse, i.e. the weight of this connection, *s* is the type of the synaptic receptor, *E*_*s*_ is the synaptic reversal potential and *τ*_*s*_ the synaptic decay time constant. At the arrival time (*t*_*i*_ + λ), a new spike propagates to the post-synaptic neuron *j*, the synaptic current jumps to the value *g*_*ij*_ and finally decays exponentially with rate *τ*.

The effect of different pairings of neurotransmitter and postsynaptic receptor can be expressed by means of combinations of (*E*_*s*_, *τ*_*s*_), with the latter representing the duration of a neurotransmitter re-uptake and dispersal. The dominant excitatory neurotransmitter in this simulation is glutamate, which corresponds to *AMPA* and *NMDA* postsynaptic receptors, while the corresponding inhibitory neurotransmitter, *γ*-Aminobutyric acid, is thought to bind to *GABA*_*A*_ receptors. Following the methodology in [19], the inhibitory receptors *GABA*_*B*_ are not explicitly simulated, since they mainly evoke intracellular signal transduction in the post-synaptic neuron instead of generating current [128].

Furthermore, certain types of synapses in the network are thought to be plastic (see Fig 1.A), in order to simulate the effect of short-term facilitation and depression found in real BG connectivity [129–131], but not simulated until recently [22]. In particular, striatal gabaergic efferents to GPe and SNr have been shown to be facilitated in periods of MSN bursts [70, 129, 130], while GPe-SNr synapses have the opposite effect [129]. The remaining SNr afferents that originate from STN have been predicted in [22] to also be depressing, a mechanism that was later found to be regulated by *GABA*_*B*_ receptors [132]. Finally, short-term effects of plasticity have been reported to exist between more structures in the BG but in some cases without a clear facilitating or depressing pattern (e.g. GP-GP recurrent synapses [130]) and in other cases very slowly activated (e.g. GPe-STN synapses [131]). Hence, these chemical synapses have been treated as fixed.

In the case of a plastic synapse, two extra variables, $u_{s}^{+}$ and $x_{s}^{-}$, are used to calculate the level of facilitation and depression respectively [133]. Their dynamics are governed by

$$\begin{array}{c} \tau_{d}\frac{dx_{s}^{-}}{dt}=1-x_{s}^{-} \end{array}$$ (6)

$$\begin{array}{c} \tau_{f}\frac{du_{s}^{+}}{dt}=U-u_{s}^{+} \end{array}$$ (7)

where *τ*_*f*_ and *τ*_*d*_ define the exponential decay time constant, and the abstract parameter *U* ∈ [0, 1] controls the amount of synaptic facilitation. At the time *t* = *t*_*i*_ + λ of a postsynaptic event, the two plasticity variables update to

$$\begin{array}{c} x_{s}^{-}\leftarrow x_{s}^{-}*\left(1-u_{s}^{+}\right) \end{array}$$ (8)

$$\begin{array}{c} u_{s}^{+}\leftarrow u_{s}^{+}+U*\left(1-u_{s}^{+}\right) \end{array}$$ (9)

with 0 < *U* < 1, and the final synaptic current that arrives at the postsynaptic neuron is given as

$$\begin{array}{c} I_{ij}^{s}\left(t_{i}+\lambda \right)=u_{s}^{+}x_{s}^{-}g_{ij}\left(E_{s}-v_{j}\right) \end{array}$$ (10)

The values for the parameters *U*, *τ*_*rec*_ and *τ*_*fac*_ can be found in [22]. Although the model of plasticity used in this study was more complex than Eqs (6–10), our synaptic models resulted to almost identical relations between synaptic IPSP amplitudes and spike frequencies as in [22] (See S4 Fig).

Finally, when two neurons *i* and *j* of the network have a direct electrical connection, or gap junction, they both receive an extra current

$$\begin{array}{c} I_{ij}^{gap}=g_{gap}\left(v_{gap}-v_{i/j}\right) \end{array}$$ (11)

where *g*_*gap*_ is the conductance (weight) of the gap junction and *v*_*gap*_ represents the potential of an extra mutual compartment at the point of the interaction [23]. This potential links the two neurons via the equation

$$\begin{array}{c} \tau_{gap}\frac{dv_{gap}}{dt}=v_{i}+v_{j}-2v_{gap} \end{array}$$ (12)

and provides a force that decreases the difference between the neuron voltages with rate *τ*_*gap*_.

All things considered, the total input *I* that a neuron receives via Eq (2) has the general form

$$\begin{array}{c} I=I^{ampa}+B\left(v\right)I^{nmda}+I^{gaba}+I^{gap}+I_{spon} \end{array}$$ (13)

where $I^{x}=\sum_{i}I_{ij}^{x}$ is the sum of all synapses of type *x*, $B\left(v\right)=\frac{1}{1.0+0.28*e^{-0.062v}}$ is the voltage-dependent magnesium plug in the NMDA receptors [23] and *I*_*spon*_ is an extra spontaneous current that is used to fit each neuron model to both *in vitro* and *in vivo* neurophysiological recordings.

#### Neuromodulation

Neurons in the BG receive dopamine from the SNc which can affect the impact of the synaptic current of certain neurons as well as the internal dynamical behaviour of others. The neurons and synapses that are affected by dopamine are depicted in Fig 1.A. Although the level of dopamine is considered to have a single fixed value throughout the system, we have used two variables *d*_1_ = *d*_2_ that correspond to the *D*1- and *D*2-like receptor families respectively, and influence the system differently.

To account for the dopaminergic effects, the synaptic input (13) as well as the neuron Eqs (2 and 3) change according to the Table 2.

**Table 2:** Neuron equations and synaptic input with dopamine.

**MSN**_*D*1_	*v*_*r*_ ← *v*_*r*_(1 + *β*_1_*d*_1_)	*β*_1_ = 0.0289
	*d* ← *d*(1 − *β*_2_*d*_1_)	*β*_2_ = 0.331
	*I*^*nmda*^(1 + *β*_3_*d*_1_) + *I*^*ampa*^ + *I*^*gaba*^	*β*_3_ = 0.5
**MSN**_*D*2_	*k* ← *k*(1 − *β*_1_*d*_2_)	*β*_1_ = 0.032
	*I*^*ampa*^(1 − *β*_2_*d*_2_) + *I*^*nmda*^ + *I*^*gaba*^	*β*_2_ = 0.3
**FSI**	*v*_*r*_ ← *v*_*r*_(1 + *β*_1_*d*_1_)	*β*_1_ = 0.1
	*I*^*ampa*^ + *I*^*gaba*^(1 − *β*_2_*d*_2_)	*β*_2_ = 0.625
**STN**	(*I*^*ampa*^ + *I*^*nmda*^)(1 − *β*_1_*d*_2_) + *I*^*gaba*^(1 − *β*_2_*d*_2_)	*β*_1,2_ = 0.5
**GPe**	(*I*^*ampa*^ + *I*^*nmda*^)(1 − *β*_1_*d*_2_) + *I*^*gaba*^(1 − *β*_2_*d*_2_)	*β*_1,2_ = 0.5

Equations and parameters are taken from [19] and [23].

#### Cortical input

The BG receive their main input from pyramidal glutamatergic projections from layer V of different areas of the cortex as well as the Thalamus [119]. Since the circuitry modelled here captures connectivity principles existing in most of the BG parallel layers [8], the main focus of the cortical simulation lies on the oscillatory nature of these inputs rather than region-dependent characteristics. Hence, thalamic input is omitted and cortical afferents are represented by abstract isolated neural ensembles *T*_*i*_, each realized through 1000 inhomogeneous poison event generators with rate parameter

$$\begin{array}{c} \lambda_{i}\left(t\right)=A_{i}cos\left(2\pi f_{i}t+\phi_{i}\right)+F_{i}^{spon} \end{array}$$ (14)

where *f*_*i*_ is the frequency, *A*_*i*_ is the amplitude, *ϕ*_*i*_ ∈ [0, 2*π*) the phase and $F_{i}^{spon}$ the tonic spontaneous firing rate of the oscillatory ensemble *T*_*i*_. Each of these ensembles is considered to project afferent axons in a single channel (with the same index *i*) of the BG circuitry, without affecting the rest of the cortical activity.

Base firing rate of a tonic cortical ensemble is thought to have a mean value of 3 spikes/sec, equal to the non-oscillating spontaneous activity ${\bar{F}}_{i}^{spon}$, while a phasically-active ensemble oscillates with amplitude *A*_*i*_ = 7 spikes/sec and thus peaks at $F_{i}^{spon}+A_{i}=10$ spikes/sec.

This behaviour is consistent with recordings in corticostriatal pyramidal cells of motor [35, 134] and sensory [135, 136] cortices, two of the regions that are greatly involved in sending excitatory inputs to the BG.

### Neural parameter estimation

Phenomenological spiking neuron models offer a computationally cheap and powerful method for neural simulations, whose accuracy, however, depends on the quality of fine-tuning of the model’s parameters. This process can be very difficult for models that contain a large number of parameters that need to be adjusted or for real neurons with a large repertoire of behaviours that need to be replicated, and for this reason various methods have been proposed (for a review see [137]). To fine-tune the neurons in GPe, STN and SNr, we employed a hybrid method, presented in [138], that combines a global and a local optimization algorithm to create models that approximate the neural behaviour recorded in empirical studies. In particular, as an objective function, we took into account the major electrophysiological properties of these neurons (e.g. the action potential amplitude and width, the resting and threshold potentials, the rheobase current, etc), as well as their steady-state frequency-current (F-I) and voltage-current (V-I) relations.

Due to great similarities of the overall BG dynamical behaviour across species, capturing which is the primary goal of this work, animal studies were taken into account in cases where human studies do not provide enough information on the electrophysiology of individual BG cells. Hence, although the resulting network may fail to capture some of the underlying physiological mechanisms, it was able to closely reproduce known rich dynamics of the neurons located in the BG nuclei, as shown in detail below.

#### GPe model

Although GPe neurons in primates have been shown to exhibit two spiking patterns (HFP and LFB neurons [69]), it is not yet clear whether the same classification holds for their electrophysiological properties, due to the lack of intracellular recordings in primate GPe [30]. This problem can be bypassed by studying the rodent globus pallidus (GP), which is believed to be homologous to the primate and human GPe [139] with retained firing patterns [140]. In [30], GP neurons were examined intracellularly, in order to draw conclusions about the analogous structure in primates, and a different three-fold classification of the GPe neurons was proposed [30, 141]. In this work, we followed the same approach and we created three different models of GPe neurons that correspond to the three different types of GP neurons in [30].

**Fig 7:**
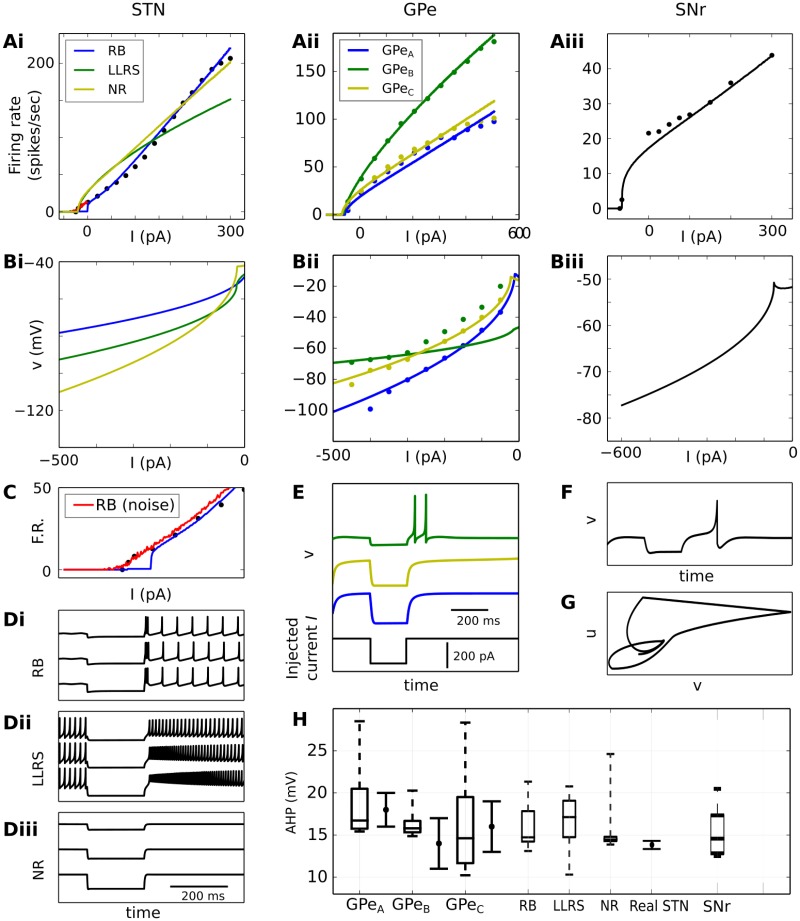
Properties of BG tuned neurons. **A**: Steady-state F-I curves of the tuned models (solid lines). **Bi-iii**: V-I curves of the same neurons. In both cases, the coloured dots represent real *in vitro* recordings. STN: Real F-I data obtained from [126] and V-I data provided by Daisuke Kase and Keiji Imoto. GPe: Real data of the three neuron types retrieved from rat slices in [30]. SNr: Real recordings were extracted from [32] and are compatible with later observed slope (12.8 + −1.13 spikes/sec per 100 pA) in [143], while the neuron’s rheobase current was taken from [144] and it is around −65 pA. **C**: Better fit of RB neurons to the real F-I curve is achieved by applying constant Gaussian noise with *σ* = 1.5 mV. **D**: Responses of STN neurons to hyperpolarizing current steps of −100, −200 and −400 pA. **E**: Responses of GPe neurons to hyperpolarizing current reveals rebound behaviour in GPe_*B*_ cells. **F**: Neuron response for hyperpolarizing current −0.6 nA matches recordings in [145]. **G**: Phase portait of the SNr neuron in (F). **H**: Box plot of the AHP amplitudes for varying capacitance *C*, along with the real mean and std for each neuron type retrieved from the same study.

Table 3 includes all intrinsic parameters and Fig 7 illustrates the basic properties of the three resulting neuron models that show distinct electrophysiological characteristics and match to the literature. From a behavioural perspective, all GPe neurons have similar rheobase currents but only type B neurons are able to evoke rebound firing (Fig 7.E). Furthermore, the firing rate of type B neurons increases almost linearly with increasing input rate, while types A and C peak at around 10 and 14 spikes/sec respectively.

**Table 3:** GPe and SNr neuron parameters.

	GPe				SNr	
Parameter	Type A	Type B	Type C	source		source
*v*_*r*_ (mV)	-50.7	-53	-54	Taken from [30]	-64.58	The value from [142] ±5
*v*_*t*_ (mV)	-42	-44	-43	Taken from [30]	-51.8	Taken from [32]
*v*_*peak*_ (mV)	38	25.0	34.5	Taken from [30]	9.8	Calculated from [32]
*C*_*fig*_ (pF)	55	68	57	Tuned manually	172.1	Tuned manually
*C*_*sim*_ (pF)	70 ± 16.5	68 ± 16.4	65 ± 16	Optimized	200 ± 44.5	Optimized
*a*	0.29	0.0045	0.42	-”-	0.113	-”-
*b*	4.26	3.895	7	-”-	11.057	-”-
*c* (mV)	-57.4	-58.36	-52	-”-	-62.7	-”-
*d*	110	0.353	166	-”-	138.4	-”-
*k*	0.06	0.943	0.099	-”-	0.7836	-”-
*I*_*vitro*_ (pA)	107	52	187.5	-”-	150	-”-
*I*_*vivo*_ (pA)	167	64	237.5	Tuned manually	235	Tuned manually
*σ* (mV)	3	3	3	-”-	5	-”-

Interestingly, the behaviour of type B neurons closely resembles the HFP cells in GPe while the other two types behave very similarly to LFB cells. Taking this into account, in this study we consider GP neurons of type B as HFP neurons found in primates and neurons of types A and C as LFB. Hence, to determine the percentage of each type of neurons in our modelled GPe, we kept the ratio of HFP:LFB found in [69] (*N*_*HFP*_ = 85% and *N*_*LFB*_ = 15%). In addition, to further break down LFB neurons into types A and C, we used an approximation based on the number of neurons examined in [30], where *n* = 14/76 and *n* = 38/76 for type A and C neurons respectively. The final ratios of GPe neurons are given in Table 1.

#### SNr model

GABAergic SNr neurons show relatively simple, agile behaviour, that can be captured by a single set of parameters. They are able to spontaneously fire high-frequency spikes that quickly turn into bursts or silence via either excitation or inhibition respectively, by the three basic BG pathways (For review see [146]). This behaviour is facilitated by their ability to emit rebound spikes [145] whose intensity changes with respect to the level of hyperpolarization. However, these cells are not able to directly influence the internal dynamics of the BG since they project only to the thalamus and dopaminergic neurons.

The majority of the electro-physiological data used for tuning the SNr neurons here were extracted from a study in rat’s SNr [32] which served as the basis of our model. Thus, data from other experimental studies were selected only if consistent with the former. The parameters of the resulting model are shown in Table 3 and its final behaviour is illustrated in Fig 7.

#### STN model

Neurons in STN can be categorized according to their response after long hyperpolarization, since they exhibit three distinctively different behaviours [53, 115, 126]. The majority type of neurons elicits short rebound bursts (RB), as a response to pallidal GABA_*A*_ inhibition [115], while a quarter of the STN neurons respond with long-lasting rebound spikes (LLRS) at lower firing rates [53]. Finally, a small amount of neurons do not produce any rebound effect and thus can be called no-rebound (NR) neurons.

When depolarized above the rheobase level, STN neurons exhibit a more homogeneous behaviour, with a sigmoid F-I relation [126, 147] and they are able to fire at high firing rates of more than 100 spikes/sec [126]. Their distinct patterns of rebound response, as well as the sigmoid shape of their F-I relation, are mainly regulated by calcium via voltage-gated calcium channels (Ca^2+^) that activate in low thresholds [53, 115, 147], as well as a type of Ca^2+^-activated K^+^ ion channels (SK K_*Ca*_ channels) [126].

Our approach here was to model the three different STN types with different sets of equations and to introduce one additional recovery variable (*u*_2_) to Izhikevich equations, as suggested in [125] and [138], to account for the effects of the aforementioned ionic mechanisms, without losing the basic repertoire of dynamical behaviours that are supported with the basic recovery variable *u* = *u*_1_.

With the addition of *u*_2_, Eqs (2–4) change to

$$\begin{array}{c} C\frac{dv}{dt}=k\left(v-v_{r}\right)\left(v-v_{t}\right)-u_{1}-w_{2}\cdot u_{2}+I+CN\left(0,\sigma^{2}\right) \end{array}$$ (15)

$$\begin{array}{c} \frac{du_{1}}{dt}=a_{1}(b_{1}\left(v-v_{r}\right)-u_{1}) \end{array}$$ (16)

$$\begin{array}{c} \frac{du_{2}}{dt}=a_{2}(Gb_{2}\left(v-v_{r2}\right)-u_{2}) \end{array}$$ (17)

For NR neurons *G* is set to be equal to 1, while for RB and LLRS neurons *G* = *H*(*v*_*r*2_ − *v*) is the heaviside step function. This makes *v*_*r*2_ to act as a threshold below which, the recovery variable *u*_2_ activates, causing rebound responses.

Furthermore, when *v* ≥ *v*_*peak*_ + *Uu*_2_, the model variables reset to

$$\begin{array}{c} v=c-Uu_{2} \end{array}$$ (18)

$$\begin{array}{c} u_{1}=u_{1}+d_{1} \end{array}$$ (19)

$$\begin{array}{c} u_{2}=u_{2}+d_{2} \end{array}$$ (20)

revealing two more mechanisms of the new recovery variable.

Besides hyperpolarization, calcium-related ion channels also activate after the rising phase of APs, influencing their shape, as well as the F-I relation of the neuron, therefore *d*_2_ ≠ 0. One of their effects, particularly visible during rebound bursts [126], is to decrease the size of the APs. In the equations above, this effect is controlled by the term *U*. Since *d*_2_ ≠ 0, the value of *u*_2_ can increase dramatically at high firing rates, causing the AP height to converge to a zero value. Hence, to avoid this phenomenon, we set

$$\begin{array}{c} U=\frac{1}{w_{1}\left|u_{2}\right|+\frac{1}{w_{1}}} \end{array}$$ (21)

which minimizes the impact of *u*_2_ to the AP size when |*u*_2_| >> 0.

Like in the case of GPe neurons, to determine the ratios of each type of neurons in our modelled STN, we used a rough approximation based on the number of neurons examined in [115]. In this study, 17 out of 20 neurons were found to elicit rebound bursts, 5 of which had a long duration and thus can be considered as LLRS neurons. The final ratios of STN neurons are given in Table 1.

The parameters of the final optimized models are shown in Table 4 and their properties are illustrated in Fig 7, where the strengths and weaknesses of each model are clear. While all neurons reproduce the rebound activity of their corresponding biological counterparts, only RB neurons were successfully tuned to follow the sigmoid pattern of the STN F-I relations. However, this was adequate to prevail the behaviour of the STN nucleus, since these neurons constitute its vast majority.

**Table 4:** STN neuron parameters.

Parameter	RB	LLRS	NR	source
*v*_*r*_(*mV*)	-56.2	-56.2	-58.5	[148]
*v*_*t*_(*mV*)	-41.4	-50	-43.75	[149]
*v*_*peak*_(*mV*)	15.4	15.4	15.4	[149]
*C*_*sim*_(*pF*)	23 ± 6.4	40 ± 8.8	30 ± 8.4	Optimized
*C*_*fig*_(*pF*)	23	40	23	-”-
*a*_1_	0.021	0.05	0.44	-”-
*b*_1_	4	0.2	-1.35	-”-
*c*(*mV*)	-47.7	-60	-52.34	-”-
*d*_1_	17.1	1	17.65	-”-
*a*_2_	0.123	0.001	0.32	-”-
*b*_2_	0.015	0.3	3.13	-”-
*d*_2_	-68.4	10	92	-”-
*v*_*r*2_ (mV)	-60	-60	-43.2	-”-
*k*	0.439	0.3	0.105	-”-
*w*_1_	0.1	0.01	0.001	-”-
*w*_2_	0	0	1	-”-
*I*_*vitro*_ (pA)	56.1	25	-1	-”-
*I*_*vivo*_ (pA)	56.1	8	-18	Tuned manually
*σ* (mV)	0.5	0.5	0.5	-”-

All things considered, neuron optimization was conducted successfully for the purposes of this study, resulting to models with realistic dynamical behaviour and electrophysiological properties. However, for a more accurate result, that focuses on the complex dynamics of individual neurons in STN, further work is required. This would involve optimization based on broader criteria, such as the distinction between transient and steady-state F-I and V-I relations, which was however impossible here, due to the lack of consistent electrophysiological data.

### Connectivity estimation

The transmission delays of impulses across the synapses of our system were taken from [19], and their values are shown in Table 5. In this section, we present the methodology we used, in the form of an algorithm, to estimate the maximum synaptic conductances *G*_*i*_ of the network, as well as two neural parameters (the external spontaneous current *I*_*spon*_ = *I*_*vivo*_ and noise *σ*) based on information about the BG connectivity and firing rate taken from the literature.

**Table 5:** Synaptic parameters.

Connection	λ (ms)	*G* (nS)	*G*_0_ (nS)	*E* (mV)	*τ* (ms)
Ctx →_*a*_ MSN_*D*1/*D*2_	10	0.6	-	0 *	6 [23, 150]
Ctx →_*n*_ MSN_*D*1/*D*2_		×0.5 [150]	-	0 *	160 [23, 150]
Ctx →_*a*_ FSI	10	0.55	-	0 [23, 150]	6 [23, 150]
Ctx →_*a*_ STN	2.5	0.388	-	0 *	2 *
Ctx →_*n*_ STN		×0.6 [151]	-	0 *	100 *
STN →_*a*_ SNr	1.5	14 **	49.5	0 *	2 *
STN →_*n*_ SNr		×0.42 [151]	20.8	0 *	100 *
STN →_*a*_ GPe	2	1.447	-	0 *	2 *
STN →_*n*_ GPe		×0.36 [151]	-	0 *	100 *
SD_1_ →_*g*_ SNr	4	4.5	156.3	-80 *	5.2 [22, 129]
SD_2_ →_*g*_ GPe	5	5.435	21.6	-65 [22]	6 [22]
GPe →_*g*_ STN	4	0.518	-	-84 [22, 152]	8 [22, 152]
GPe →_*g*_ SNr	3	93	603.9	-80 *	2.1 [22, 129]
GPe→_*g*_ GPe	1	0.765	-	-65 [22]	5 [22]
SNr→_*g*_ SNr	1	0.2	-	-80 *	3 *
MSN→_*g*_ MSN	1	0.75 [23, 121]	-	-60 [23, 150]	4 [23, 150]
FSI→_*g*_ FSI	1	1.1 [23, 124]	-	-60 [23, 150]	4 [23, 150]
FSI →_*g*_ MSN_*D*1/*D*2_	1	3.75 [23]	-	-60 [23, 150]	4 [23, 150]

* General value for this parameter [127].

** Local optimization.

Values of *G* without explanation were obtained with manual optimization.

The arrows →_*X*_ represent synapses that express the neurotransmitter AMPA, NMDA and GABA_*A*_, for *X* = *a*, *n* and *g* respectively.

The initial value of the noise factor *σ* needed to be increased significantly for neurons in the MSN, FSI and STN, in order to simulate the effect of the different inputs to the BG from external structures that are not modelled here (e.g. other areas of the cortex). Also, a similar increase was necessary for GPe and SNr neurons, to account for inputs from other areas of STN which might correspond to different tonically-active microscopic channels. Finally, the spontaneous current *I*_*vivo*_ was also altered for each BG nucleus, in order to approximate their basal firing rates, when all synaptic inputs are blocked.

This process consisted of the following steps, that are specialized for each afferent structure and aim to approximate results of empirical experiments.

#### NMDA:AMPA ratios

Initially, the ratio of the two neurotransmitters used to model the glutamatergic synapses of our model needed to be determined for all excitatory synapses shown in Fig 1. As discussed in [112] and [23], it has been shown that FSI neurons receive only AMPA excitatory input from the cortex. Götz et al. [151] investigated the effect of AMPA and NMDA receptors in the rest of the glutamate-based synapses of the BG and found that they both play an important role in the excitation of the BG neurons. To approximate the NMDA:AMPA conductance ratios, we considered the ratios of the peak current for each type of receptor, which was obtained in [151] using glutamate in nucleated patches of BG cells. The final values for each ratio are given in Table 5. Hence, to estimate connectivity weights of the excitatory synapses we tuned only one conductance (AMPA) which was used to infer the corresponding NMDA values.

#### Striatum

This was the first structure whose connectivity was tuned, since its activity does not depend on any other BG nuclei according to our model’s architecture. The dominant striatal cell, the MSN, fires at 0.01–2.0 spikes/sec in basal tonic mode and 17–48 spikes/sec in periods of high activation or bursting [22, 153]. Also, *in vivo* mouse recordings have found that the basal firing rate FSIs in the striatum is between 10–15 spikes/sec while it increases up to 60–80 spikes/sec during behavioral tasks [154, 155].

To tune our striatal neurons, we employed only the model of striatum, striatal afferents and internal striatal connectivity, we initially set all cortical firing rates to be 3 spikes/sec and then we changed *T*_1_ to 10 spikes/sec to account for tonic and bursting modes respectively. The parameters that were tuned are *σ*_*msn*_, *σ*_*fsi*_, *G*_*ctx*−*msn*_ and *G*_*ctx*−*fsi*_.

#### STN

The basal firing rate of STN is around 10 spikes/sec and increases 100% without the influence of GPe [31]. In periods of high activation, STN neurons show mixed dynamical behaviour and fire at around 30–50 spikes/sec [15]. Hence, to tune the network properties of STN, we followed the next two steps:

1. Similarly to the previous case, we used only the model of STN and tuned parameters related to cortical afferent axons (*G*_*ctx*−*stn*_, *σ*_*stn*_ and *P*_*ctx*−*stn*_) in order to make it fire at around 20 spikes/sec in tonic mode (without GPe inhibition) and around 40 spikes/sec in periods of high activation.
2. We then forced GPe to fire at 30 spikes/sec (by using a poisson process instead of the neuron equations) and tuned *G*_*gpe*−*stn*_ to make STN fire at around 10 spikes/sec.

An adequate result was achieved by setting the conductance strength of the cortico-striatal afferents, for both AMPA and NMDA receptors to 0.25 nS, decreasing the STN noise to *σ*_*stn*_ = 0.5 mV and setting *P*_*ctx*−*stn*_ = 3% which results to 30 spikes/sec arriving to each STN neuron in the tonic mode and 100 spikes/sec in periods of high activation.

#### GPe

Recordings of the GPe have shown that its basal firing rate is around 30 spikes/sec [22]. After STN lesions, GPe’s activity decreases 50% [156] while it increases 55% without striatal inhibition and local collaterals [157]. The parameters that influence the basal firing rates and connections between STN and GPe and need to be tuned are *I*_*vivo*−*gpe*_, *G*_*stn*−*gpe*_, *G*_*msn*−*gpe*_, *G*_*gpe*−*gpe*_ and *σ*_*gpe*_. The first parameter has been already optimized in order to make each type of GPe neurons to be close to the critical state between their two firing modes (see Neural parameter estimation). Since the remaining four-dimensional parameter space is complex for hand-tuning, we employed the classical Nelder-Mead method for local search [158], with the following fitness function:

1. Use GPe (without the Striatum and local collaterals) and force STN to fire at 10 spikes/sec. Return |*FR*(*GPe*) − 46.5|.
2. Turn the striatum and local GPe collaterals on and return |*FR*(*GPe*) − 30|.
3. Turn STN off and return |*FR*(*GPe*) − 15|.

#### SNr

Different reports show SNr to fire at rates between 22–29 spikes/sec, when the BG operate normally (STN at 10, the striatum around 1 and the GPe around 30 spikes/sec) [22, 159–161]. Also, without the influence of the GPe, SNr is shown to increase its firing rate more than 300% [22, 157], while without STN, the firing rate is decreased 50% [156].

To approximate the effect of the incoming synapses to SNr, we used again the local search method described above, in the parameter space {*I*_*vivo*−*snr*_, *G*_*stn*−*snr*_, *G*_*snr*−*snr*_, *G*_*msn*−*snr*_}. The fitness function in this case includes the following steps:

1. Turn off GPe, and reduce maximum conductance of STN-SNr connections to *G*_*stn*−*snr*_/2. Return |*FR*(*SNr*) − 76.5|.
2. Turn off STN and force GPe to fire at 15 spikes/sec. Return |*FR*(*SNr*) − 12, 5|.

The two-fold reduction of STN maximum conductances was necessary to simulate the effect of the depressive STN synapses to SNr [162], since its firing rate will be increased 100% without the influence of GPe. As the final step, using the whole BG model, we hand-tuned *G*_*gpe*−*snr*_ such that SNr fires at around 25.5 spikes/sec, which is the average value of the different findings.

#### Short-term plasticity

The above procedure results in a static model of the BG connectivity, where the strength of all synapses remains fixed for the whole duration of a simulation. Its behaviour represents the stead-state tonic mode of the BG circuit, where synaptic conductances *G* have already been modulated with respect to the tonic firing rate of the pre-synaptic neuclei.

To find the initial conductance of each synapse *G*_0_, we need to calculate the degree by which it changes in tonic mode. If *STF*_*X*_(*f*) encodes the conductance change due to short-term facilitation for a nucleus *X* and firing rate *f*, and *STD*_*X*_(*f*) the corresponding relation for depression, then the current synaptic conductance can be found as $G_{0}^{X}\left(f\right)=G^{X}/STF_{X}\left(f\right)$ or $G_{0}^{X}\left(f\right)=G^{X}/STD_{X}\left(f\right)\cdot U$ for facilitating or depressing synapses respectively. Hence, from S4 Fig: *STF*_*SD*1_(1.1) = 1.5, *STF*_*SD*2_(1.1) = 1.05, *STD*_*GPe*_(30) = 0.154 and *STD*_*STN*_(10) = 0.283. The final conductances *G*_0_ are given in Table 5.

In conclusion, estimating connectivity between and within the BG nuclei comprises a semi-automated procedure that resulted in a model with realistic firing rates in both tonic mode and during periods of high cortical activation (Fig 1.B and 1.C). This procedure should be followed again, in case that a different number of channels or neurons within a channel is chosen.

## Supporting information

S1 Fig Connectivity of the STN in the phasic mode. The STN sends diffuse connections to the GPe that spread across all simulated neighbouring channels. The transparency of the arrows represents the firing rate of the source structure.
(EPS)

S2 Fig Transfer entropy for various delays. Animated visualization of transfer entropy between the BG structures and the cortex for different values of the time delay of information flow. SD1/2: MSN neurons with d1/2 dopamine receptors respectively, T2: Phasically-active cortical ensemble.
(GIF)

S3 Fig Impact of the cortical phase offset to the TE of GPe and STN in different frequency ranges. Inner circle: Normalized TE of GPe (**A**) and STN (**B**) afferents versus efferents across phase offsets *ϕ*. Outer circle: Normalized TE of the indirect (**A**), hyper-direct (**B**) and direct (**C**) pathways as defined in Fig 5.
(EPS)

S4 Fig Effect of short-term plasticity in synaptic conductances. Ratio between steady-state conductance *G* and the initial value *G*_0_ for different pre-synaptic spike frequencies, and for all plastic connections of the BG circuit.
(EPS)
