# Supplementary figures and images for: The role of cortical oscillations in a spiking neural network model of the basal ganglia
Source: PLoS One. 2017 Dec 13;12(12):e0189109. doi: 10.1371/journal.pone.0189109 (PMC5728518; doi:10.1371/journal.pone.0189109)

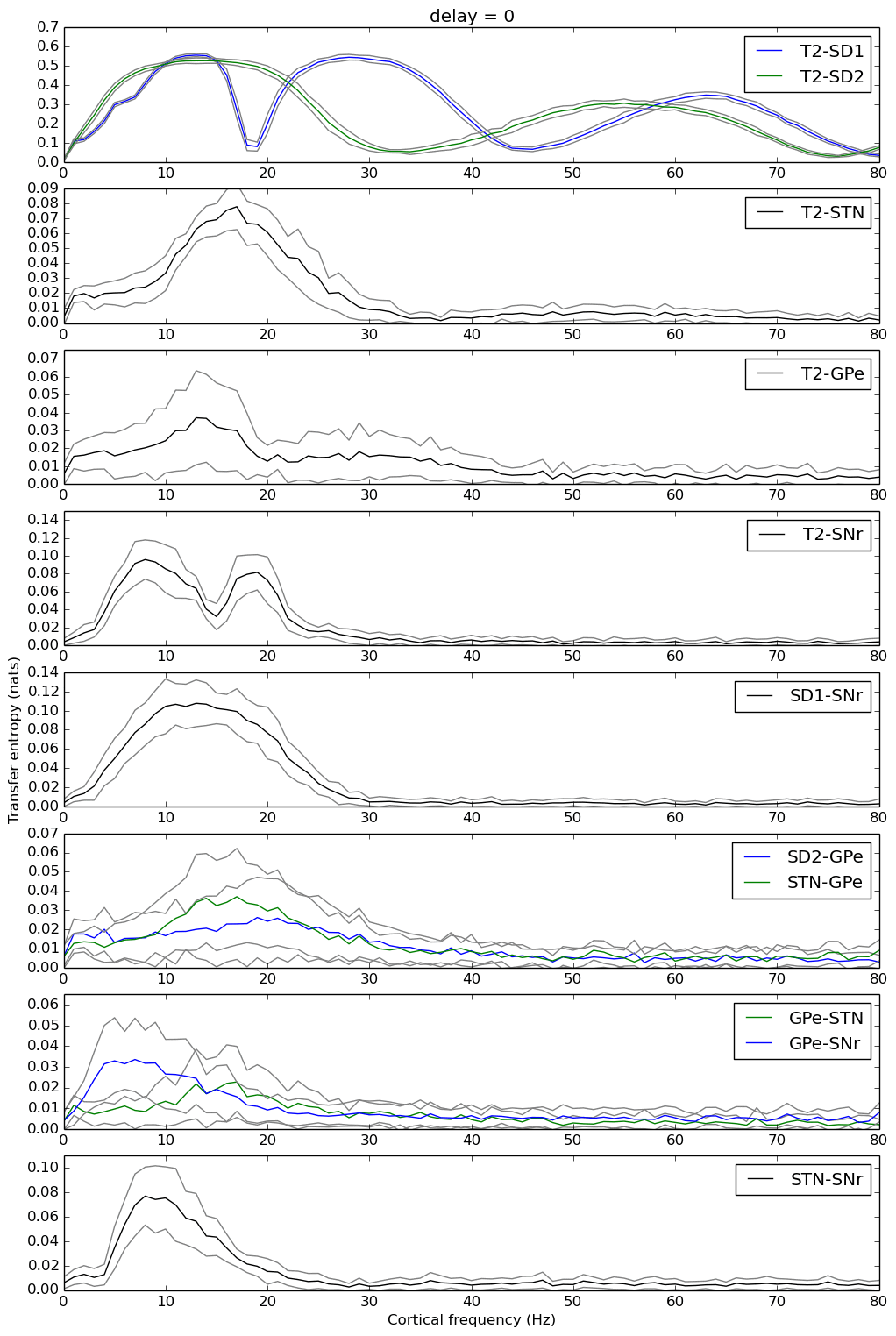

Supplement: S2 Fig — Animated visualization of transfer entropy between the BG structures and the cortex for different values of the time delay of information flow. SD1/2: MSN neurons with d1/2 dopamine receptors respectively, T2: Phasically-active cortical ensemble. (GIF) [file pone.0189109.s002.gif]
