# Supplementary material for: Correction: Correction: The role of cortical oscillations in a spiking neural network model of the basal ganglia
Source: PLoS One. 2020 Apr 22;15(4):e0232314. doi: 10.1371/journal.pone.0232314 (PMC7176110; doi:10.1371/journal.pone.0232314)
Supplement: S1 File — (PDF) [file pone.0232314.s001.pdf]

PLOS ONE

CORRECTION

Correction: The role of cortical oscillations in a spiking neural network model of the basal ganglia

Zafeirios Fountas, Murray Shanahan

The image for Fig 1 is incorrect. The correct version is provided here. The figure caption is correct.

Check for updates

OPEN ACCESS

Citation: Fountas Z, Shanahan M (2018) Correction: The role of cortical oscillations in a spiking neural network model of the basal ganglia. PLoS ONE 13(10): e0205472. https://doi.org/10.1371/journal.pone.0205472

Published: October 29, 2018

Copyright: © 2018 Fountas, Shanahan. This is an open access article distributed under the terms of the Creative Commons Attribution License, which permits unrestricted use, distribution, and reproduction in any medium, provided the original author and source are credited.

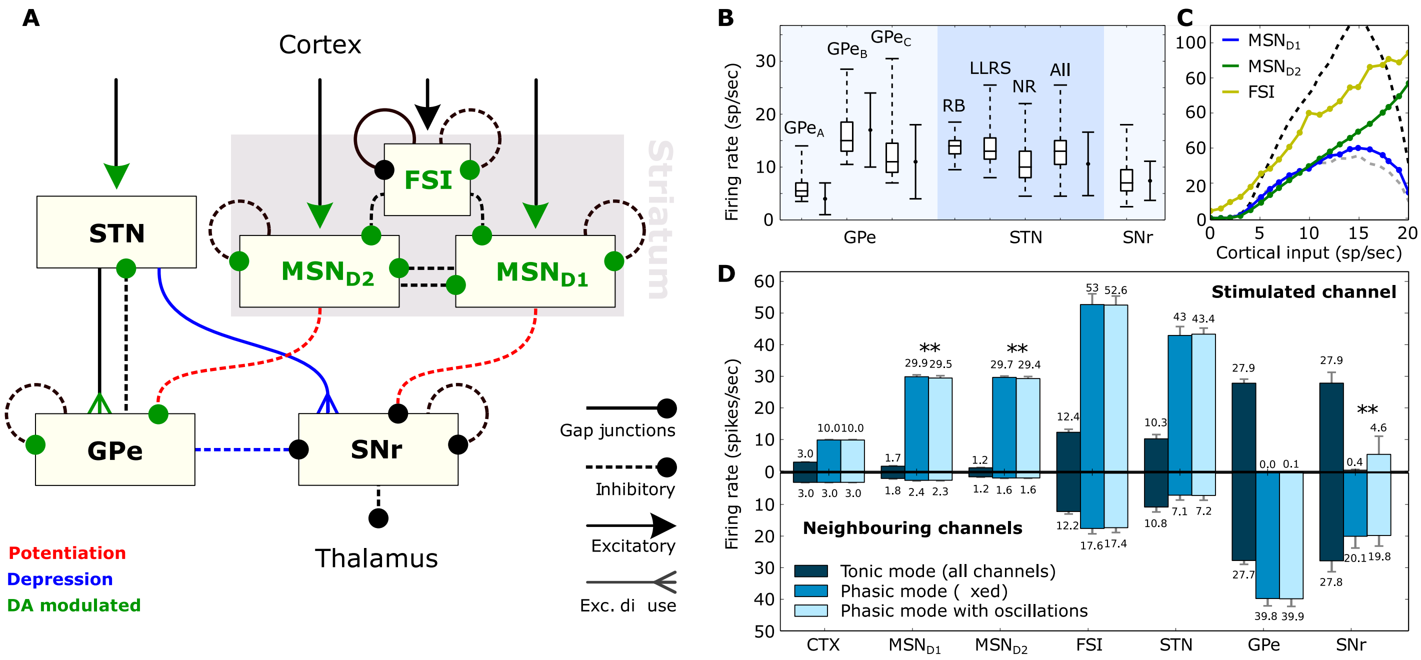

Fig 1. Architecture and firing behaviour of the system. A: The BG circuit as realized in the present study. Dopamine (DA) influences both the internal behaviour of MSNs and FSIs as well as the impact of various synaptic conductances. B: Firing rates of the various neuron types when isolated. Boxplots show median, first-third quartiles, and minimum-maximum of the mean firing rate of each simulated neuron. Solid error bars represent mean and standard deviation of recorded (real) spontaneous activity. Data for GPe neurons were taken from [30], for STN neurons from [31] and for SNr neurons from [32]. Neuron sub-types of the striatum include medium spiny-projection neurons (MSN) and fast-spiking interneurons (FSI), the STN includes rebound-bursting (RB), long-lasting rebound spikes (LLRS) and no-rebound (NR) neurons, and the GPe includes the three types GPe$_{A-C}$. C: Cortical input-firing rate curve of striatal neurons when the complete model is in use. The dashed lines illustrate the MSN$_{D1}$ curve for low dopamine (grey) and high dopamine (black) in the system. D: The mean firing rates, over 500 3-second trials, of the various neuron types when the complete model is in use. The *stimulated channel* represents the channel that received enhanced cortical input during the phasic mode, while the set of bar charts below show the firing rates of the two *neighbouring channels*. The error bars show standard deviation. In tonic mode, there is no discrimination between channels and the small differences in the two sets of bar charts are the result of random noise. The double asterisk (**) denotes an independent two-sample T-test with p-value $< < 0.01$.

https://doi.org/10.1371/journal.pone.0205472.g001

Reference

1. Fountas Z, Shanahan M (2017) The role of cortical oscillations in a spiking neural network model of the basal ganglia. PLoS ONE 12(12): e0189109. https://doi.org/10.1371/journal.pone.0189109 PMID: 29236724
